# Development of an intervention to facilitate implementation and uptake of diabetic retinopathy screening

**DOI:** 10.1186/s13012-020-00982-4

**Published:** 2020-05-19

**Authors:** Fiona Riordan, Emmy Racine, Eunice T. Phillip, Colin Bradley, Fabiana Lorencatto, Mark Murphy, Aileen Murphy, John Browne, Susan M. Smith, Patricia M. Kearney, Sheena M. McHugh

**Affiliations:** 1 School of Public Health, University College Cork, Western Gateway Building, Western Rd, Cork, Ireland; 2 Department of General Practice, University College Cork, Cork, Ireland; 3 Centre for Behaviour Change, University College London, London, England; 4 Department of General Practice, Royal College of Surgeons of Ireland, Dublin, Ireland; 5 Department of Economics, Cork University Business School, University College Cork, Cork, Ireland

**Keywords:** Retinal Screening, Family Practitioner, Implementation Intervention, Intervention development, Theoretical Domains Framework, Patient and Public Involvement (PPI), Stakeholder consultation

## Abstract

**Background:**

‘Implementation interventions’ refer to methods used to enhance the adoption and implementation of clinical interventions such as diabetic retinopathy screening (DRS). DRS is effective, yet uptake is often suboptimal. Despite most routine management taking place in primary care and the central role of health care professionals (HCP) in referring to DRS, few interventions have been developed for primary care. We aimed to develop a multifaceted intervention targeting both professionals and patients to improve DRS uptake as an example of a systematic development process combining theory, stakeholder involvement, and evidence.

**Methods:**

First, we identified target behaviours through an audit in primary care of screening attendance. Second, we interviewed patients (*n* = 47) and HCP (*n* = 30), to identify determinants of uptake using the Theoretical Domains Framework, mapping these to behaviour change techniques (BCTs) to develop intervention content. Thirdly, we conducted semi-structured consensus groups with stakeholders, specifically users of the intervention, i.e. patients (*n* = 15) and HCPs (*n* = 16), regarding the feasibility, acceptability, and local relevance of selected BCTs and potential delivery modes. We consulted representatives from the national DRS programme to check intervention ‘fit’ with existing processes. We applied the APEASE criteria (affordability, practicability, effectiveness, acceptability, side effects, and equity) to select the final intervention components, drawing on findings from the previous steps, and a rapid evidence review of operationalised BCT effectiveness.

**Results:**

We identified potentially modifiable target behaviours at the patient (consent, attendance) and professional (registration) level. Patient barriers to consent/attendance included confusion between screening and routine eye checks, and fear of a negative result. Enablers included a recommendation from friends/family or professionals and recognising screening importance. Professional barriers to registration included the time to register patients and a lack of readily available information on uptake in their local area/practice. Most operationalised BCTs were acceptable to patients and HCPs while the response to feasibility varied. After considering APEASE, the core intervention, incorporating a range of BCTs, involved audit/feedback, electronic prompts targeting professionals, HCP-endorsed reminders (face-to-face, by phone and letter), and an information leaflet for patients.

**Conclusions:**

Using the example of an intervention to improve DRS uptake, this study illustrates an approach to integrate theory with user involvement. This process highlighted tensions between theory-informed and stakeholder suggestions, and the need to apply the Theoretical Domains Framework (TDF)/BCT structure flexibly. The final intervention draws on the trusted professional-patient relationship, leveraging existing services to enhance implementation of the DRS programme. Intervention feasibility in primary care will be evaluated in a randomised cluster pilot trial.

Contributions to the literature

- Interventions to improve DRS uptake demonstrate wide heterogeneity in effectiveness and limited use of explicit behaviour change strategies.
- Although routine management of type 2 diabetes largely takes place in primary care, few interventions have focused on primary care, and targeted *both* professionals and patients.
- Few studies explicitly document the process of user involvement and how it influences intervention content and delivery.
- To address these gaps, we adopted an explicit process of involving intervention users (professionals and people with diabetes) and developed an intervention for delivery in primary care, clearly outlining our development process which combines behaviour change theory, stakeholder involvement, and existing evidence of intervention effectiveness.

## Background

The number of people with diabetes is rising globally, placing a burden on health systems, people with diabetes, and their families [1]. Diabetic retinopathy (DR) is the most common microvascular complication of diabetes [2, 3]. Worldwide, it is estimated that approximately 28 million individuals have vision-threatening retinopathy [4]. In Ireland, DR affects 8.2% of the population with type 2 diabetes over 50 years (approximately 10,000 people) [5] and is one of the leading causes of blindness among adults of working age [6]. Regular diabetic retinopathy screening (DRS) leads to the earlier detection of retinopathy and treatment that can prevent or delay the development of diabetes-related blindness [7–9]. In most countries that have screening programmes, DRS is recommended annually [10–12].

Although DRS is found to be effective, uptake is often suboptimal [13–18]. In Ireland, RetinaScreen provides free, annual retinal screening (and if necessary, treatment) to anyone aged 12 years or older with diagnosed diabetes. Uptake of this government-funded population-based DRS programme, introduced in 2013, is currently 56% [19]. In the international literature, non-attendance at screening has been linked to a number of factors including younger age [14, 16], lower socioeconomic status (SES) [20–23], longer diabetes duration, type of diabetes (people with type 1 are less likely to attend) [20], and poorer glycaemic control [24]. Barriers include a lack of awareness of DR and the risk of retinopathy [24, 25], the accessibility of screening centres, and time constraints [25]. Recommendation to attend screening from a primary care health care professional (primary care HCP) encourages attendance [24–26].

‘Implementation interventions’ refer to methods used to enhance the adoption and implementation of clinical interventions such as DRS [27]. To be most effective, implementation interventions should target multiple levels [28–31]: (1) introducing system-level change to facilitate sustainability and integration with existing infrastructure, (2) providing support to health care professionals to change work practices, and (3) targeting, or intervening with, patients to change behaviours and outcomes [32]. Type 2 diabetes, which accounts for about 90% of all cases of diabetes [33], is largely managed in primary care, so it is an appropriate setting in which to introduce implementation interventions to target DRS uptake. Primary care HCPs have a role in referring to DRS services and promoting attendance. It also presents an opportunity to target patients who have stopped attending specialist care (e.g. type 1) who may have poorer diabetes control and thus be at higher risk of complications such as retinopathy [34]. Despite this, few interventions have focussed on primary care [13] and targeted *both* professionals and patients [13, 35–39].

Successful interventions to improve DRS uptake [40–42] include patient education to increase awareness of DR [35, 43, 44], patient reminders [13, 36, 37, 39, 45–53], guidelines [35], education [36, 37], or registration and reminder systems to support professionals to follow-up patients [13, 37–39, 41, 54]. However, a recent Cochrane review found that while interventions target some important barriers, they incorporate a narrow range of behaviour change techniques, with ‘missed opportunities’ to target some of the individual, social, cultural, and environmental barriers and enablers of screening attendance [55]. In addition, the effects of interventions vary widely, and this variation remains largely unexplained [40]. Few studies are explicit in terms of the frameworks and theories used to guide intervention development [13, 44, 56]. Therefore, DRS is one clinical service which would benefit from a more systematic, theory-based approach to improve implementation.

There is ongoing uncertainty about the best approach to develop and tailor interventions [30, 57, 58]. Despite mixed evidence on the contribution of theory to intervention effectiveness [59, 60], it is a central part of many approaches to developing interventions. User involvement is recommended as another key component [61, 62] to tailor the content of interventions to context (i.e. primary care setting) [30] and align with stakeholder preferences [63]. Despite recognition that these elements are important, the challenge is how to combine these elements, for example, it is often unclear how development moves from theory to decisions about intervention content, format, and delivery, and what role stakeholders play in this step [64]. There is a need for case examples which clearly outline all steps of the development process, in particular how to utilise theory while also eliciting and integrating the perspectives of end users, drawing on elements of coproduction.

## Aim

Our aim was to use a systematic process combining theory, stakeholder involvement, and existing evidence of the effectiveness of interventions, to develop a multifaceted implementation intervention targeting both primary care HCPs and patients to improve the update of DRS. A broader aim of this paper is to provide a case study of how to systematically develop an implementation intervention drawing on both theory and stakeholder involvement, and to highlight some of the challenges and lessons inherent in this approach.

## Methods

### Design

The IDEAs (Improving Diabetes Eye screening Attendance) intervention was developed by combining theory, stakeholder involvement, and evidence [58, 65]. By stakeholders, we mean people with diabetes, professionals, and representatives from the national screening programme. We drew on the principles of co-creation, defined by Leask et al. as ‘collaborative public health intervention development by academics working alongside other stakeholders’ [66], along with the INVOLVE definitions of collaboration ‘an ongoing partnership between you and the members of the public you are working with, where decisions about the research are shared’, and consultation ‘when you ask members of the public for their views and use these views to inform your decision-making’ [67]. Stakeholder involvement was part of an overall effort to co-create the intervention; specifically, involvement comprised collaboration with HCPs and patients (users of the intervention) over the course of the development process, and consultation with representatives from the national screening programme to ensure integration of intervention components with existing processes.

**Fig. 1:**
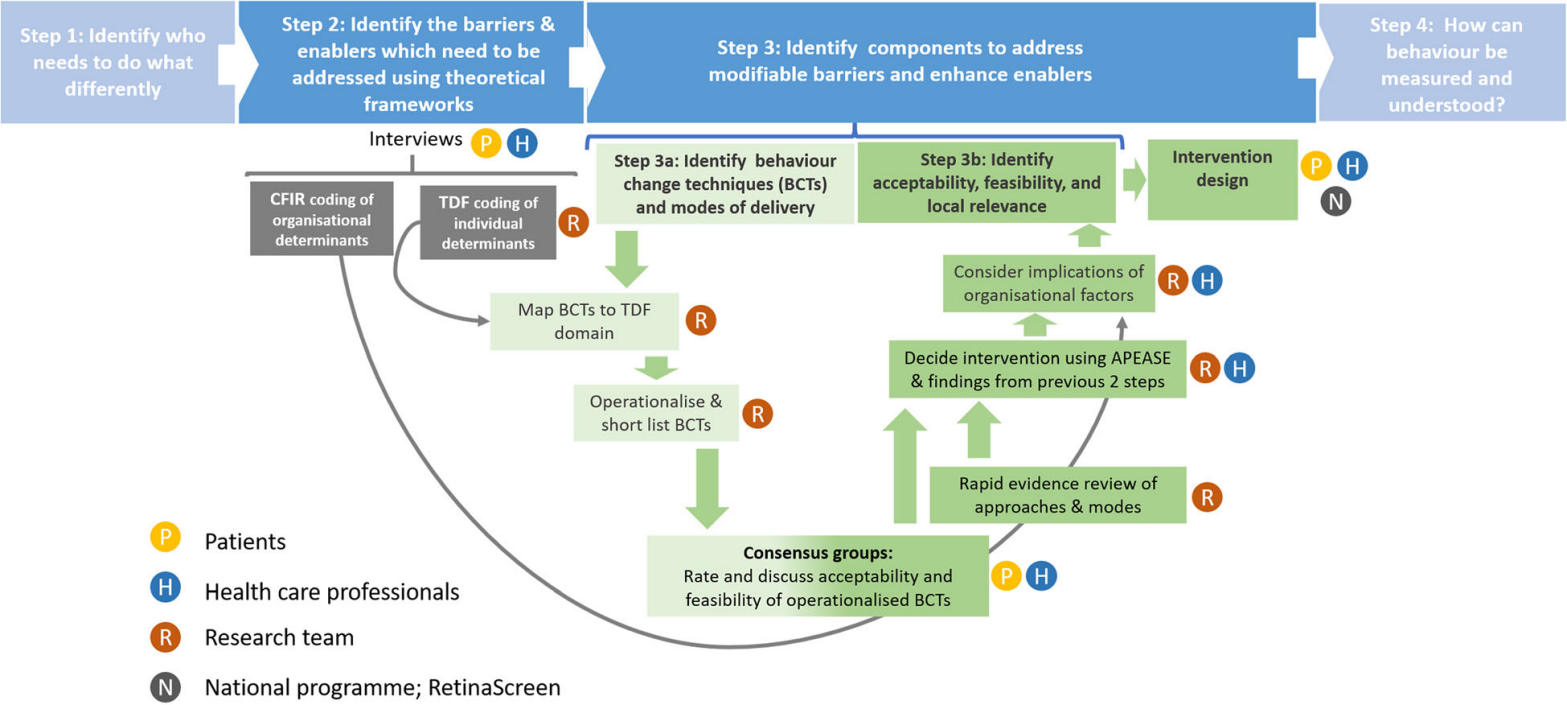
Overview of the development process. TDF, Theoretical Domains Framework; DRS, Diabetic Retinopathy Screening; BCT, behaviour change technique; CFIR, Consolidated Framework for Implementation Research

The core intervention development work took place in 2018/2019 when the national screening programme had begun to introduce new approaches to facilitate participation [68]. This stepped systematic development process [65] has been used to develop implementation interventions in different settings [69, 70] (Fig. 1). The final intervention was reported according to guidance from TiDieR [71] and Proctor et al. [27].

#### Step 1: Identify who needs to do what, differently

DRS involves a number of potential different behaviours being performed by different individuals. We therefore aimed to identify which behaviours to target by analysing data from an existing audit of screening uptake in two large primary care centres in the South of Ireland [68].

#### Step 2: Identify the barriers and enablers to be addressed using theoretical frameworks

To identify barriers and enablers, we analysed existing qualitative data collected as part of a study to understand patient and professional experiences of DRS among other diabetes services. Patient interviews were conducted by MT (July 2014 and January 2015) (see “Acknowledgements”). Participants were purposively sampled from the list of audited patients according to screening status (i.e. attenders, non-attenders, non-consenters) defined according to the audit. Participants were not sampled by other demographic factors*.* Patient interviews were conducted at the two primary care centres [72]. A semi-structured interview guide was used to explore patient knowledge of diabetes and DR and their history of attending existing DRS services and experience of RetinaScreen, including the reasons for deciding to participate (or not) in the new programme (Additional file 1).

HCP interviews and focus groups were conducted by FR and KON (PhD researchers in the team at the time—see “Acknowledgements”) between April 2016 and February 2017. HCP participants were purposively sampled according to their role and region of Ireland, not other demographic factors*.* Interviews with GPs, PNs, and DNS were conducted in a mix of general practices across Ireland (GPs, PNs), health administrative offices (DNS), and hotels (DNS) to coincide with professional conferences. HCP interviews were conducted as part of a broader realist evaluation to understand the implementation of the national clinical programme for diabetes, including the establishment of a national DRS programme [73]. As such, the topic guide was informed by attendance patterns and initial theories about how the DRS programme was working. It also included open questions to elicit HCPs’ experiences of engaging with the national DRS programme, for example, barriers to, and facilitators of, the registration process (Additional file 1). Interviews were digitally recorded, transcribed verbatim, and imported into NVivo 10 software for analysis.

The analytical approach for this intervention development study did not follow the principles of realist analysis. Instead, the deductive analysis was directed by the Theoretical Domains Framework (TDF) and informed by a coding structure developed as part of a recent systematic review of screening attendance (Additional file 2: Table S1) [25] which was used to code interviews to identify the barriers and enablers to the target behaviours and to guide the choice of intervention components [74]. In instances where a single TDF domain did not apply, multiple domains were applied. Barriers and enablers were compared with those identified in the systematic review [75]. Two members of the research team (FR and SMH) prioritised factors which were identified in both international and Irish contexts, and the Irish context only, and focused on *modifiable* factors, i.e. those which would be possible to address through an intervention delivered through primary care. *Non-modifiable* factors (i.e. system-level factors which were beyond the scope of this primary care intervention) were shared with the national programme stakeholders.

The Consolidated Framework for Implementation Research (CFIR) was also used to code the HCP interviews to ensure organisational level influences were thoroughly considered, specifically, to identify if they could be targeted by the intervention or to identify non-modifiable factors which might act as wider implementation determinants. These determinants of uptake were considered during the next step of the development process.

#### Step 3: Identify and decide the intervention components to address modifiable barriers and enhance enablers

##### 3 (a) Identify behaviour change techniques and modes of delivery

Barriers and enablers, grouped by theoretical domains, were mapped to appropriate behaviour change techniques (BCTs). The mapping process was based on published expert consensus about effectiveness for behaviour change [76, 77] and a ‘TDF matrix’ constructed by Lawrenson et al. as part of their Cochrane review of interventions to improve DRS uptake [40]. The matrix links BCTs to TDF domains, indicating which techniques are likely to be effective in changing that particular domain. The mapping process resulted in a long list of potential BCTs for the intervention.

The initial long list of BCTs was narrowed down based on whether the technique had been shown in other studies to be effective as part of interventions to increase DRS [40] or if there was evidence in the wider literature on public health interventions of effectiveness of the technique in other settings (e.g. smoking cessation, interventions to improve diet and exercise). BCTs were retained if specific examples of how to operationalise them to improve DRS uptake were available [40, 55].

Operationalised BCTs were further refined following review by members of the research team (FR, SMH, SMS, PMK, JB, AM) together with the expert input of a behavioural scientist (FL), while also considering the scope of the current study, and non-modifiable organisational factors. For example, operationalised BCTs completely beyond the scope of the study, such as ones requiring changes to the operation of the national retinopathy screening programme were excluded at this point. Remaining BCTs were revisited to ensure we had at least included ones which corresponded to salient TDF domains, that is those frequently mentioned and/or deemed to be of high importance by the researchers or participants.

##### 3 (b) Identify feasibility, local relevance, and acceptability of the intervention

*Consensus groups*

Three semi-structured consensus group meetings were held with people with diabetes (meeting 1), people with diabetes *and* HCP (meeting 2), and HCP only (meeting 3) to discuss the feasibility of proposed intervention components (operationalised BCTs) and suitable modes of delivery. Further details on the consensus group meetings, including recruitment of patients and members of the public, are provided as part of a SWAT (Study Within A Trial) which observed group dynamics and gathered data on participants’ experiences of the meetings [78]. Before the meetings, participants were given a short summary of evidence on barriers and enablers of DRS attendance and approaches to address non-attendance. Participants were also sent an electronic or paper survey, to assess feasibility and acceptability, based on a validated instrument developed by Weiner et al. [79] (Additional file 3). Participants were asked to rate the acceptability and feasibility of each component on a five-point Likert scale ranging from ‘strongly disagree’ to ‘strongly agree’. Consensus meetings were facilitated by an experienced facilitator (JB). During the meetings, a summary of the survey results was presented to participants followed by a series of small group discussions facilitated by FR, SMH, and EP. Participants were asked to consider how each component would work in practice and which mode of delivery would work best.

*Populating the APEASE criteria*

The final components were decided by a subgroup of the research team (FR, SMH, PMK, SMS) and a GP collaborator (MM) based on the APEASE (affordability, practicability, effectiveness, acceptability, side effects, equity, sustainability) criteria. Acceptability and practicability criteria were populated with findings from consensus meetings using a process informed by previous studies which utilised consensus methods [80] (Table 1). The effectiveness criterion was based on a rapid evidence review of different approaches to improve screening (e.g. text/letter/phone reminders and messages, educational materials, brief interventions and narrative leaflets) (see Additional file 4: PubMed search strategy). The remaining criteria were based on group discussions among the subgroup of research team and GP collaborator.

**Table 1:** Overview of the shortlisting process

If ≥ 70% of participants agreed* the component was acceptable it was retained. *If < 70% of participants agreed the component was acceptable*: (1) If all three groups discussed the component in the meetings… • If all were either, unsure whether it was feasible *or* felt it was unfeasible or unacceptable then it was excluded. • If two groups felt it was feasible and one did not then this component was considered. • If only one group felt it was feasible then this component was excluded. (2) If only two groups discussed the component… • If one group felt it was unfeasible or unacceptable and the other did not then group composition was considered. For example, if professionals in the mixed group or professional-only group expressed concerns about feasibility, then this was given more weight than if concern was expressed by people with diabetes felt the component was feasible in primary care. (3) If only one or no groups discussed the component… • If < 70% participants agreed it was feasible then this component was excluded. If ≥ 70% agreed it was feasible then this component was included.	

* Survey response categories were collapsed into ‘Agree’, ‘Neither agree nor disagree’ and ‘Disagree’

After this, the research team deliberated on the available evidence for each shortlisted component and the proposed mode of delivery. We considered organisational factors identified through patient interviews (i.e. at the level of the screening programme),and HCP interviews (i.e. at the level of the practice) coded using CFIR. If non-modifiable, the team considered how best to work around/with this factor to help the fit of the intervention components within the primary care environment. Following a decision on the final intervention components, RetinaScreen were consulted to determine whether additional modifications were needed. Intervention study materials were prepared by a graphic designer, reviewed by the National Adult Literacy Agency (NALA) and a Patient and Public Involvement (PPI) group established to incorporate additional patient involvement in developing the intervention materials and advise on the study procedures on an ongoing basis. Materials were subsequently revised to include their suggestions.

#### Step 4: How can behaviour change be measured and understood?

A logic model of the final IDEAs intervention, representing the inputs, processes, and the causal mechanisms by which it is expected to achieve change was developed, in conjunction with deciding the feasibility of outcome measures for the pilot trial.

Ethical approval was obtained from the Clinical Research Ethics Committee for the Cork Teaching Hospitals.

## Results

### Step 1: Identify who needs to do what, differently

The audit findings highlighted suboptimal uptake and distinguished gaps at the level of the professional and patient, specifically, registration with the programme (professional), consent for the programme to hold their contact details and send them an appointment letter (patient), and attendance once they received their appointment (patient) [68]. Hence, it was decided the intervention should target both people with diabetes and professionals.

### Step 2: Identify the barriers and enablers to be addressed using theoretical frameworks

In addition to the 47 patient interviews (Additional file 5: Table S2), 22 interviews were conducted with (GPs (*n* = 5), practice nurses (*n* = 9), and DNS (*n* = 8), and 2 focus groups (4 per group) were conducted with community DNS (*n* = 8).

Examples of barriers for patients included confusion between screening and routine eye checks (‘Knowledge’), forgetting (‘Memory, attention, decision processes’), and anticipation of a negative result (‘Beliefs about consequences’). Enablers included a recommendation from friends/family or HCPs (‘Social Influences’). HCP barriers included the time to register patients which was impeded or supported by practice resources (‘Environmental context and resources’). HCPs also lacked information on screening uptake in their local area (‘Knowledge’). *Modifiable* patient and professional-level factors mapped to BCTs are provided in Additional file 6: Table S3 (a and b).

Several organisational factors, identified both in the interviews and the Cochrane review and classified using CFIR, were not modifiable within the scope of the study and reflected structural and organisational aspects of the screening programme (e.g. accessibility of screening centres, being able to reschedule appointment, competing demands such as getting time off work, presence or absence of media coverage to increase awareness) (Additional file 7: Table S4).

### Step 3: Identify and decide intervention components to address modifiable barriers and enhance enablers

#### 3(a) Identify potential behaviour change techniques and modes of delivery

Selected operationalised BCTs within each domain (*n* = 48) were organised according to whether they operate at the patient, professional, or organisational level, i.e. features of the wider practice context which impede or enable professionals to register patients. Some operationalised BCTs deemed to be outside of scope of the study were excluded, for example, sending a congratulatory letter to attendees (requiring the input of RetinaScreen) or supporting patients to develop a plan for how often they will attend screening, where it will take place and how they will get their appointment (requiring extensive input from primary care professionals in a face-to-face consultation). Shortlisted operationalised BCTs are indicated in Additional file 8: Table S5 (a, b and c) along with reasons for exclusion.

#### 3 (b): Identify feasibility, acceptability and local relevance of the intervention

Consensus groups

In total, 16 patients and 15 HCPs, including GPs, practice nurses, diabetes nurse specialists, and an ophthalmologist, took part in the consensus process. Most of the 39 proposed components (operationalised BCTs) were acceptable to ≥ 70% of participants, while the response to feasibility varied (Additional file 9: Fig. S1). For example, while 80% of participants felt it would be acceptable to identify someone in the practice to help the patient to register and consent, only 60% felt this would be feasible.

During consensus meetings, some components were supported across all groups. Providing feedback on screening uptake to practices was considered essential. Delivering reminders and messages through a GP or nurse was favoured, albeit some professionals were uncertain about the feasibility of communicating messages to patients given how ‘stretched’ resources are in primary care.

I think someone in the practice [should deliver the message], again your GP, with a good solid kind of relationship with your GP, I think you will take more notice of him or her (Patient, Group 1)

Participants deemed certain components unfeasible or inappropriate. For example, arranging social support was considered a potential breach of confidentiality and only appropriate in some very specific circumstances. In other instances, there were divergent opinions among people with diabetes and HCPs. For example, HCPs believed publicising the numbers who attended at their practice could work for some but not all patients; however, patients disagreed:

If you say, sure 95% of people go to their screening. Aw sure, I dont need to go so. I don't want to be shamed or I don’t want to feel like I am being shamed (Patient, Group 1)

Where groups reflected on the actual content of specific messages, they emphasized the need to avoid ‘scare tactics’ while also being able to ‘dispel a false sense of security’. Participants suggested some system-level changes which were unfeasible within the scope of the current study, for example, provision of alternative opening hours or greater data sharing between RetinaScreen and primary care practices.

### Populating the APEASE criteria

We considered the implications of organisational factors, classified using the TDF and CFIR, integrating this step into our final decision process using APEASE (Table 2). This led us to introduce certain intervention components to accommodate the additional organisational challenges, to encourage participation, aid the roll out of the intervention, and enhance some enablers, but these components were not *necessary* or core for the intervention to work. For example, reimbursement and technical assistance were added as a new staff resource could not be introduced to conduct the audit. We also included components to mediate the effect of certain non-modifiable factors (e.g. informing patients about the ease with which they can reschedule their appointment, recognising that patients have competing demands and that out-of-hours screening could not be provided within the study) (Additional file 7: Table S4). We also refined delivery modes at this point. For example, a practice briefing was added as a way to communicate important messages (BCTs) to professionals.

**Table 2:** Final decision process using APEASE

Component	Decision ✓ included ✗ excluded	Who	When	Rationale for inclusion based on *APEASE* criteria
Briefing/training on the intervention	**✓**	–	–	• To overcome organisational barriers.
Audit and feedback	**✓**	Practice nurse	At baseline and at six months	• Component demonstrated *effectiveness*. • Advise practices that nurse should conduct audit if *practical* but allow practices to decide locally.
Reimbursement	**✓**	–	–	• To overcome organisational barriers. Should be considered reimbursement rather than incentive as asking practices to do something extra not doing existing work better
Electronic prompt	**✓**	Administrator	Every appointment	• Component demonstrated *effectiveness*. • Based on *practicality*, advise practices that administrator should add prompts but allow practices to decide locally. • Alerts cannot be added selectively to the type of patient consultation (i.e. review vs. opportunistic) therefore occurring at every appointment based on *practicality*. • Alert fatigue could be an issue if patient has many alerts on their file.
Information leaflet				
Enhanced/Endorsed	**✓**	GP or practice nurse	By post, and opportunistically in appointment also	• Component demonstrated *effectiveness* and is *practical.* • GP/Nurse most *effective* as represent BCT (‘Information from a credible source’). Delivery modes *practical* within general practice.
Narrative	✗	–	–	• Personal narrative not *practical*. To be most *effective* requires several different iterations (ages, gender, stage of disease). Generic narrative may not be *acceptable* to all. • GP/Nurse most *effective* as represent BCT (‘Information from a credible source’).
Reminder message				
Face to face	**✓**	GP or practice nurse	Part of every consultation; prompted by electronic alert on patient file	• Component demonstrated *effectiveness* and is *practical.* • GP/Nurse most *effective* as represent BCT (‘Information from a credible source’). Delivery modes *practical* within general practice.
Phone call	**✓**	Practice nurse	–	• Component demonstrated *effectiveness* and is *practical.* Delivery mode is *sustainable* within general practice as fits within existing practices, and *equitable* as possible to reach most patients using this approach. • Nurse most *practical* and *effective* as represents BCT (‘Information from a credible source’). First considered administrative staff making the phone calls; however, if there is a script and probing it becomes an education opportunity and makes more sense for practice nurse to deliver this component.
Follow-up letter	**✓**	GP	–	• Component demonstrated *effectiveness* and is *practical*. Delivery mode is *sustainable* within general practice as fits within existing practices, and *equitable* as possible to reach most patients using this approach.
Text message	✗	–	*–*	• Confidentiality (*safety*) concerns, that is, the wrong individual could access and read text messages). Not all patients may own or use mobile phones, as such, the mode may not be *equitable*. Not all practices use this mode and will have established acceptable consent processes, therefore not *practical*.

After considering the consensus group findings and evidence of effectiveness, in terms of the APEASE criteria, the components of the final IDEAs intervention included a practice briefing, audit and feedback with technical support, practice-endorsed reminders (delivered in person, by phone and letter), and an information leaflet targeting key attitudinal and knowledge barriers (Table 3). Following consultation with the national DRS programme, we decided to include the self-registration and consent form with the GP-endorsed letter and information leaflet, to provide patients with another way to participate in the programme.

**Table 3:** Final intervention components mapped to the barrier or enabler to be targeted, TDF or CFIR domain in which the barrier or enabler operates, BCT (s)

Barrier (–) or enabler (+)	TDF/*CFIR*	BCT	Operationalised components mapped to *implementation strategies* [76]
(–) HCP lack knowledge on service uptake in their practice	Knowledge *Access to information and knowledge*	Feedback on behaviour	***Audit and provide feedback*** Practice audit of patients with diabetes
			***Conduct educational meeting (briefing)*** Added as a delivery mode for HCP messages*
(+) HCP feel that patient attendance at the screening programme facilitates follow-up care with patients and means patients can access screening closer to home	Beliefs about consequences *Relative advantage*	Info. about social and environmental consequences	Explain registering and ensuring patient attends the programme will mean they know their patients’ status and patients will be able to get routine care closer to home
**(+)** HCP register patients and encourage attendance because they believe in quality diabetes care	Social prof. role/identity *Compatibility*	Framing/reframing	Encourage HCPs to see influencing patients to attend as part of their role in delivering good diabetes care
(–) The process of checking the register/registering patients (online or via phone) is lengthy and too resource intensive	Environ. context *Complexity*	-	***Provide technical support*** Added to accommodate the fact a new staff resource could not be introduced to conduct the audit 1. Audit and intervention manual and laminated reminder script for face-to-face or phone encounters 2. Researcher-led training for the staff member conducting audit
(+) HCP who attended diabetes courses know that registering patients to attend screening is part of their role	Knowledge Memory, attention, decision processes (MADP) *Access to information and knowledge*	Prompts/cues Restructuring physical environment	***Remind clinicians*** Electronic prompts
(+–) Length of time taken to register patients or to check the register; patient registration depends on practice resources (–) HCPs felt there was ‘no money’ in tracking/encouraging patient attendance	Environ. context *External policies and incentives*	-	***Use other payment schemes*** Reimbursement
			***Intervene with patients***
(–) Patients forget to respond to the consent letter and/or forget appointment	MADP	Prompts/cues	**Reminder messages** (face-to-face, phone or letter) in which GP/nurse…
(+) HCP recommendation	Social influences	Social support (unspecified) Providing info. about others’ approval Credible source	…provides general encouragement to attend appointment
(–) Patients find it difficult to consent via phone process (+) HCPs support patient attendance: they register patients, check registration, facilitate consent	Skills Environ. context	Social support (practical) Restructuring the social environ.	…advises people how they can arrange appointment and specify they should ask GP/nurse for help if needed
			**Information leaflet which…**
(–) Patients find it difficult to consent via phone	Skills	Instruction how to perform the behaviour	…explains how they can arrange an appointment^║^
(–) Patients confused between screening and routine eye tests; think they do not need to attend the new screening programme	MADP Knowledge	Info. on health consequences	…clarifies difference between screening and routine checks, that screening is part of their care, and routine checks are not a substitute^║^
(+) Some patients recognised screening as a routine part of their diabetes care	MADP Knowledge	Framing/reframing	…encourages patients to adopt a different perspective on retinal screening; not extra but part of routine/optimal self-management
(–) Patients do not have symptoms (–) Patients do not link symptoms to diabetes (–) Patients unaware of the link between diabetes and eye damage (–) Patients confident they are not at risk	MADP Knowledge Emotion	Salience of consequences Info. about health consequences	…communicates information about risk; (1) that *everyone* with diabetes is at risk, (2) there may be no symptoms, and (3) screening applies to them too^║^
(–+) Some patients are disengaged with diabetes care while others are in a routine of going for tests and feel ownership or responsibility over their diabetes	Beliefs about capabilities Social prof. role/identity	Verbal persuasion to boost self-efficacy	…encourages them to charge of their health
(–+) Some patients believe screening service is ‘looking for money’ while others know the service is free	Beliefs about consequences Environ. context *External policies and incentives*	Info. on social and environmental consequences	…emphasises the programme is free^║^
(–) Some patients lack awareness of the importance of screening or generally do not perceive the necessity of screening (+) Others believe screening provides valuable information on their eye health status, facilitates early detection of problems and provides reassurance or consequences are salient; patients have experienced complications or know others who have (–) Patients anticipate negative outcome of screening and fear a bad result (–) Patients are afraid of the harmful effect of screening procedure (+) Fear or anxiety about vision loss	Beliefs about consequences Beliefs about consequences Emotion	Info. about health consequences Info. about emotional consequences	…reassures patients that after their appointment they will be fine or can be treated early to stop things getting worse^║^
(–) Patients dislike the drops administered during appointments	Environ. context *Compatibility*	Non-modifiable	…emphasises the effects of the drops are short term vs. the benefits of screening
(+) Service flexibility, the ability to ring the programme and reschedule appointment helped people to attend (–) Competing demands (e.g. unable to take time off work or have family dependents)	Environ. context *External policies and incentives*	Non-modifiable	…highlights it is possible to reschedule their appointment to a time which suits them best.

*Environ. context* environmental context, *Social prof.* role/identity, social professional role and identity, *MADP* Memory, attention and decision processes

*Some components were added to accommodate the additional organisational challenges, to encourage participation, aid the roll out of the intervention, and enhance some enablers but are not *necessary* or core for the intervention to work

^║^Also included as part of face-to-face reminder message

### Intervention design

Following review by NALA and the PPI panel, some changes were made to the wording of the messages and the overall design of the intervention materials. For example, we reduced the amount of information provided on the instruction page of the information leaflet and emphasised ‘friendliness’ and helpfulness of RetinaScreen staff. RetinaScreen gave permission to use their logo to make the information leaflet potentially more familiar to patients if they had already received materials from the programme. It also ensures both patients and HCPs are aware the intervention aligns with the national programme. Materials were also designed using a colour scheme similar to that used by RetinaScreen (Additional files 10, 11 and 12).

### Step 4: How can behaviour change be measured and understood?

#### Logic model

**Fig. 2:**
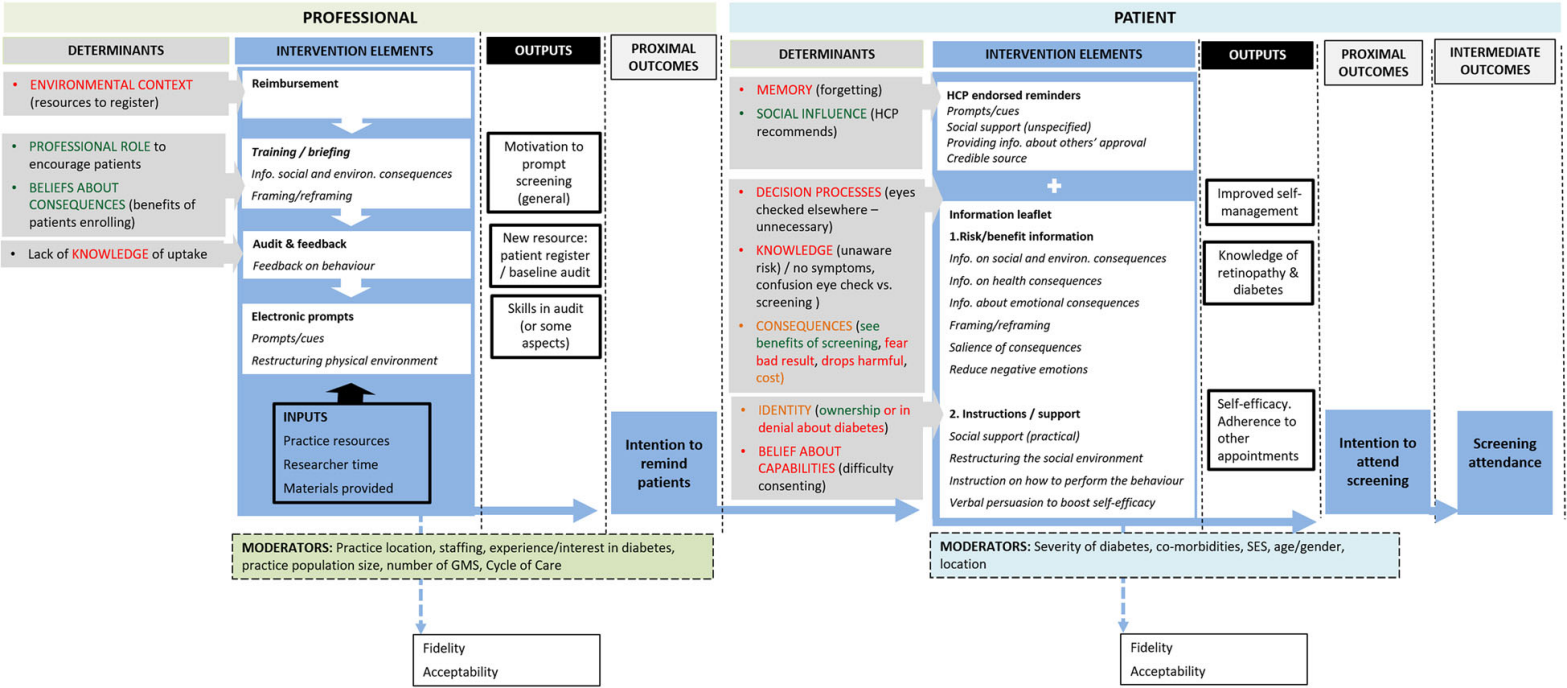
Logic model of the professional and patient-level intervention mapped to determinants (barriers and enablers according to the Theoretical Domains Framework) and BCTs to improve screening attendance

The intervention is expected to work by enabling professional behaviour change (financial resource) and improving their knowledge of non-attendance. Patient behaviour is expected to change by using HCPs to prompt and persuade patients to attend screening, altering beliefs or attitudes about the consequences of screening, and enhancing self-efficacy by offering support and information about how to participate in screening (Fig. 2). Relevant theories which may explain the mechanism of action, include the Health Belief Model [81], Protection Motivation Theory [82], the Theory of Planned Behaviour [83] (patient behaviour), and control theory [84] (professional behaviour), along with macro level theories, Systems Theory, Institutional Theory, and Contingency Theory [85]. These will be explored further through the process evaluation embedded in the feasibility trial of the intervention [86].

## Discussion

This paper outlines the development of a multifaceted, multi-level implementation intervention using a systematic process combining theory, collaboration with multiple stakeholders, and existing evidence of effectiveness of interventions, using the case of screening uptake as an example. Enhancing the implementation of screening programmes is an important issue not only in Ireland but internationally, as evidenced by suboptimal uptake reported in different countries [13–16]. Given the efficacy of screening, and the resources invested in these programmes, it is important to maximise attendance. Non-attendance is also costly; within a UK primary care organisation, missed DRS appointments were retrospectively calculated to cost £78,259 per annum [87]. To improve DRS uptake, our intervention will target both professional behaviour (using practice briefing, training, reimbursement, audit and feedback, and prompts) and patient behaviours (using practice-endorsed reminder messages and information leaflet). Our process outlines how to develop a theory-driven intervention while involving stakeholders throughout and integrating their perspectives and preferences.

To our knowledge, our intervention to improve DRS uptake is one of few targeting both professionals and patients to be delivered through primary care. The advent of international and national reforms to strengthen care delivery in the community means the role of primary care in chronic disease management is increasingly important. Primary care HCPs have an ongoing contact with people with diabetes and may have the potential to reach vulnerable patients who have stopped attending specialist care [88]. GP endorsement has been used effectively to increase uptake of cancer screening [89–91]. The current intervention serves as an example of how the doctor-patient relationship [24, 25], and existing local services (i.e. primary care practices), may be leveraged to support the implementation of population-based programmes.

### Strengths and limitations of the study

This paper clearly documents the development process and the decisions on intervention content and mode of delivery. We outline how BCTs were operationalised to form intervention components. While the APEASE criteria are often used to contextualise an intervention, few studies apply it systematically [92]. We faced initial challenges when applying the criteria. We surveyed members of the research team asking them to rate each shortlisted component based on APEASE. However, we found they were less comfortable applying some criteria, for example, effectiveness. To address this, we carried out a rapid evidence review and conducted an open discussion whereby members were informed of the evidence and could contribute their different expertise to different criteria. We specified the data sources which were ultimately used to populate each criterion and inform our decision on intervention components. A further strength is the multidisciplinary collaborative approach to develop the intervention. In line with MRC recommendations [62], we ensured that end users, namely people with diabetes and primary care HCPs, were involved at different steps throughout the study. Firstly, interviews with people with diabetes and HCP interviews were used to identify barriers and enablers specific to the Irish context. As these were consistent with factors identified in the international review, we are confident that the intervention targets salient barriers and enablers. Secondly, a two-stage consensus process involved professionals and patients in co-creating the intervention, paying particular attention to how components would work in real world practice. Thirdly, our research team is multidisciplinary blending expertise in implementation research and practice, comprising academic GPs and ophthalmologist, health services researchers, an epidemiologist, economist, and behavioural scientist. Consultation with the national DRS programme ensured the feasibility and ‘fit’ of the intervention with existing processes. Lastly, we established a PPI panel, which enabled patients to be further involved in finalising the study materials. A final strength is that our intervention incorporates a number of BCTs identified as ‘missed opportunities’ by the Cochrane review, that is, BCTs which theoretically mapped to important barriers but were infrequently used in existing approaches to improve uptake [55]. Specifically, we included BCTs which addressed emotional barriers to screening attendance such as fear of a bad result following screening.

The intervention has some limitations. The intervention is delivered through primary care and largely relies on endorsement from primary care HCPs. While patients with type 2 diabetes should attend their GP regularly, some individuals may have infrequent or no contact with their primary care provider [93], for example, patients who are disengaged with health services and self-management more generally, due to diabetes-specific distress [94] and the phenomenon of ‘diabetes burnout’ [95]. Furthermore, not all patients may heed messages delivered through their GP, instead favouring messages communicated by friends or family. Support from family members was an enabler identified both in the international literature [25] and in our patient interviews. The influence of family and friends may be possible to emulate through personal, narrative-based leaflets. There is some evidence they can increase intention to attend [96], improve attitudes towards [97], and increase uptake of colorectal screening [98]. While a narrative approach was proposed as an element of the IDEAs intervention, this was ultimately discounted as there were mixed opinions about the most feasible mode of delivery and whether a generic narrative would be acceptable and effective for different groups of patients. Given the diverse profile of people with diabetes that multiple versions (photos, messages) of any leaflet may be important to enhance effectiveness, this approach may be more feasible when the target demographic is reasonably homogenous.

The intervention may need to be adapted for certain population subgroups. As part of the process evaluation, we will pay due attention to fidelity and adaptations (e.g. delivery mode) and whether patients considered the intervention appropriate for them. For example, while NALA reviewed our study materials such that we confident they are readable and accessible for the general population, we acknowledge this intervention does not address language barriers and literacy challenges in population subgroups. Language was an issue identified in the Cochrane review [25]. In Ireland, over 18% of adults (and 28% of adults aged 55–65) are at or below Level 1 on the literacy scale [99].

Certain factors may affect how feasible it is for our implementation intervention to be applied in this context. Primary care is diverse in terms of team composition, organisational structure, size, and workflow [100]. Time constraints and workload [101], physician communication style [102], competencies and knowledge [101, 102], protocols to structure care [101], and unclear division of labour [101, 103] may present barriers to implementation.

That some suggestions fell outside the scope of our study indicates there may be a need for broader, system-level changes to address some of the prevailing challenges in relation to screening attendance (e.g. accessibility/transport and the limited reach of information campaigns). Our intervention does simulate some system-level changes, but at the level of primary care practices, e.g. audit and feedback. As part of the pilot trial, practices will record the reason patients did not attend screening [86]. This information, together with qualitative research with patients as part of the process evaluation, will allow us to explore factors which potentially moderate the effect of the intervention (see examples Fig. 2). It may allow us to explore the reasons for different behaviours, including outstanding issues with screening attendance which are not addressed by the current intervention.

### Implications

We identified a number of challenges during the development process which have broader methodological relevance for implementation science, namely, challenges with respect to applying the TDF/BCT structure; challenges using coproduction (i.e. tensions between theory-informed suggestions and those suggested by stakeholders); the utility of using both TDF and CFIR; and the potential and feasibility of applying the APEASE criteria systematically. These elements are relevant for those developing interventions beyond the DRS context.

While application of the TDF and subsequent mapping to BCTs was useful to identify theory-informed approaches to target barriers and facilitators, this process was not always straightforward. In some cases, the TDF/BCT structure needed to be applied flexibly. For example, HCP interviews were used to inform the ‘Skills’ domain at the *patient* level (i.e. patients were not always able to contact or register with the screening programme due to poor IT literacy). Sometimes, multiple TDF domains applied to a specific barrier or facilitator, whereupon multiple domains were applied. For example, patients who recognised screening as a routine part of their diabetes care would attend; this facilitator could be coded as ‘Memory and decision processes’, ‘Knowledge’, or ‘Goals’. There were instances where BCTs deemed appropriate to target a barrier or facilitator did not necessarily map to the corresponding TDF domain. Drawing on the input of researchers and academic GPs, in conjunction with an evidence review, was key to enable the TDF/BCT framework to be applied flexibly and determine which operationalised BCTs ‘made sense’. Mapping to TDF domains yielded several possible BCTs, but not all BCTs were relevant, applicable or appropriate for the behaviour in question and context of interest. In this scenario, we found the stepped process of stakeholder involvement was essential to move beyond the TDF/BCT matrix and to help us make decisions about intervention components which would not be worth pursuing based on acceptability and feasibility concerns. Ultimately, while it was useful to draw on theory and the structure afforded by TDF/BCT, in order to ground our initial intervention components and select the content and delivery mode relevant for the specific context (i.e. primary care), it was crucial to bear the real world context for the intervention and the users in mind.

Our approach also highlights potential challenges of utilising coproduction in intervention development. To involve people with diabetes and HCPs, we used a two-stage consensus approach, collecting both quantitative and qualitative data and using a validated instrument to assess acceptability and feasibility [79]. To avoid overly influencing our participants, discussions were semi-structured. This allowed them to reflect on the acceptability and feasibility of a long list of potential operationalised BCTs. However, this format also presented tensions at different steps of the process. Firstly, some end users made suggestions which fell outside of study scope or were not evidence-based; this may have reflected the fact participants were not constrained by a more structured format such as Nominal Group Technique or Delphi [104]. As suggested by Powell et al., there is value in presenting stakeholders with evidence-based options and asking them to supplement these based on their own expertise [30]. Deciding how to manage conflicts between, and prioritise, different sources of knowledge (e.g. evidence review vs. tacit knowledge and preferences of stakeholders) is recognised as important during intervention design [64]. However, there is little guidance about how best to balance these different sources. Secondly, integrating the contributions of the different groups across our process was a key challenge, particularly as professional and patient preferences did not always align. Ultimately, we resolved to make evidence-based decisions, incorporating a final check to ensure we addressed salient theoretical domains (barriers and facilitators), and weighing the contributions of patients and professionals according to the nature of their feedback, for example, professional feedback on issues of feasibility (e.g. reminder *delivery mode*), and patient feedback on issues of acceptability (reminder *messages*) were given more weight. Given some of the challenges highlighted by the current study, future studies should consider how to structure and sequence user involvement. This could be done by incorporating final ‘checks’ of user suggestions to check whether these are in line with theory or existing evidence or seeking the input of users on theory-informed intervention elements that have been designed by researchers. Future studies should consider how best to strike a balance between (a) thoroughness of end-user involvement whereby open selection from a long list of suggestions gives users more scope to shape and co-create the intervention and (b) the efficiency of using a more structured approach such as providing specific examples of interventions and asking for feedback [105].

A plethora of frameworks exist for intervention development and investigating behavioural influences [58, 106]. Our study drew on the relative strengths of different frameworks for different purposes. As in other studies [70], we found utilising CFIR together with the TDF useful to elaborate on implementation determinants in the outer and inner setting which fall within the TDF domain ‘environmental context’ and help translate barriers and enablers into practical approaches to implementation [107]. Factors not addressed by our intervention may moderate intervention effectiveness; we will use this information to guide the type of data we collect as part of the process evaluation. Our approach highlights the value of recording organisational factors.

Our study demonstrates the potential and feasibility of applying the APEASE framework in a systematic way. That is, since members of the research team lacked specific knowledge about effectiveness of intervention components and delivery modes, we conducted a rapid evidence review, allowing the team to focus on other criteria (affordability, equity, sustainability) where their expertise was most valuable.

## Conclusion

This paper outlines a comprehensive process involving intervention users to develop a multifaceted, multi-level implementation intervention to improve the uptake of a national DRS programme. By systematically applying theory, collaborating with multiple stakeholders and reviewing the evidence base, we are confident we have developed an intervention which is more likely to be feasible to deliver in primary care and acceptable to both professionals and patients. We have used the example of an intervention to improve DRS uptake to illustrate an approach to integrate theory with user involvement and some of the associated challenges. Our final intervention is designed to fit within the primary care practice workflow, leveraging the trusted professional-patient relationship and familiarity of local services to enhance implementation of a national population-level screening programme. Though developed using robust methods, the effectiveness of the intervention is not guaranteed. The feasibility of the intervention and study procedures will be assessed as part of a pilot cluster randomised trial with a view to progressing to a definitive trial. This will ultimately determine whether IDEAs is a clinically and cost effective intervention to enhance the implementation of a national DRS programme and improve health outcomes for patients with diabetes.

## Supplementary information

**Additional file 1.** Topic guides.

**Additional file 2 Table S1.** Theoretical Domains Framework: definitions and coding structure developed by Graham-Rowe et al. [26].

**Additional file 3.** Questionnaire.

**Additional file 4.** PubMed search strategy.

**Additional file 5: Table S2.** Patient characteristics.

**Additional file 6: Table S3.** a. Modifiable patient-level barriers and enablers and corresponding TDF mapped to BCTs. b. Modifiable professional-level barriers and enablers and corresponding TDF mapped to BCTs based on published expert consensus about effectiveness for behaviour change [5, 6] and the mapping matrix constructed by Lawrenson et al. [7]. b Modifiable professional-level barriers and enablers and corresponding TDF mapped to BCTs based on published expert consensus about effectiveness for behaviour change (1, 2) and the mapping matrix constructed by Lawrenson et al HTA. (3) a Modifiable patient-level barriers and enablers and corresponding TDF mapped to BCTs based on published expert consensus about effectiveness for behaviour chang e[5, 6] and the mapping matrix constructed by Lawrenson et al. [7]. b Modifiable professional-level barriers and enablers and corresponding TDF mapped to BCTs based on published expert consensus about effectiveness for behaviour change (1, 2) and the mapping matrix constructed by Lawrenson et al. HTA (3).

**Additional file 7: Table S4.** Organisational factors identified from health professional and patient interviews, coded according to CFIR and TDF.

**Additional file 8: Table S5.** a Patient-level barriers or enablers mapped to TDF, BCT and operationalisation. b Professional-level barriers or enablers mapped to TDF, BCT and operationalisation. c. Organisational level barriers or enablers mapped to TDF, BCT and operationalisation.

**Additional file 9: Figure S1.** a Ways to encourage patients to attend (practice led). b Ways to encourage patients to attend (narrative led). c Ways to encourage patients to attend (other ideas). d Ways to encourage professionals to prompt patients about screening (feedback). e Ways to encourage professionals to prompt patients about screening (feedback). f Ways to encourage professionals to prompt patients about screening (other ideas).

**Additional file 10.** Script.

**Additional file 11.** Reminder letter.

**Additional file 12.** Patient information leaflet.

## Data Availability

Not applicable.

## Abbreviations

BCTs: Behaviour change techniques
CFIR: Consolidated Framework for Implementation Research
DR: Diabetic retinopathy
DRS: Diabetic retinopathy screening
GP: General practitioner
HCP: Health care professional
IDEAs: Improving Diabetes Eye screening Attendance
MADP: Memory, attention, decision processes
TDF: Theoretical Domains Framework
