# Correction to: Development of an intervention to facilitate implementation and uptake of diabetic retinopathy screening

**DOI:** 10.1186/s13012-020-01026-7

**Published:** 2020-07-30

**Authors:** Fiona Riordan, Emmy Racine, Eunice T. Phillip, Colin Bradley, Fabiana Lorencatto, Mark Murphy, Aileen Murphy, John Browne, Susan M. Smith, Patricia M. Kearney, Sheena M. McHugh

**Affiliations:** 1 School of Public Health, University College Cork, Western Gateway Building, Western Rd, Cork, Ireland; 2 Department of General Practice, University College Cork, Cork, Ireland; 3 Centre for Behaviour Change, University College London, London, England; 4 Department of General Practice, Royal College of Surgeons of Ireland, Dublin, Ireland; 5 Department of Economics, Cork University Business School, University College Cork, Cork, Ireland

**Correction to: Implementation Sci (2020) 15:34**

**https://doi.org/10.1186/s13012-020-00982-4**

Following the publication of the original article [1], the authors realised there were two errors in Additional file 9: Figure S1. The file should have included a footnote clarifying that the total number of people who completed questionnaires was 30 (13 patients and 17 health care professionals). ‘Suppl. Figure 1 (d) – Feasible’ was a copy of ‘Suppl. Figure 1 (e) – Feasible’. The correct file has been included with this correction article.

## Supplementary information

**Additional file 9.**
