# Supplementary material for: Development of an intervention to facilitate implementation and uptake of diabetic retinopathy screening
Source: Implement Sci. 2020 May 19;15:34. doi: 10.1186/s13012-020-00982-4 (PMC7236930; doi:10.1186/s13012-020-00982-4)
Supplement: Supplementary file 1 — Additional file 1. Topic guides. [file 13012_2020_982_MOESM1_ESM.docx]

**PATIENT TOPIC GUIDE**

- Thank participant, introduce researcher & study
- Outline information sheet, consent form, highlight permission to record interview

| **Topic** | **Prompt/probe** |
| --- | --- |
| Tell me a bit about yourself | Live here always?  Family  Occupation  General health/ other health conditions?  When were you diagnosed with diabetes?  How is it at the moment?  Have you had any problems or complications in the past? |
| Have you ever had your eyes screened for diabetic retinopathy?  Had you heard of the national retinal screening programme (DiabeticRetinaScreen)?  When did you receive the invitation to the national retinal screening programme?  Do you know why you were invited to attend the screening programme?  What did you think/feel when you received the letter/ invitation?  And what happened after you received the letter? | When? Where? How often?  If yes: why?  If no: why not?  Who told you about it?  GP? Other healthcare provider? Advertising campaign?  Surprised? Confused? Had GP informed patient that they had been put on register? |
| **Attenders**  How was your screening appointment?  How long were you waiting for your results?  How did it compare to previous visits to (the previous service they attended if that was the case)  Why did you decide to go for screening? | Waiting times? Staff? Procedure (eye drops)  Advice from GP/friends/other HCP?  Free service?  Convenience? |
| **Non attenders**  Why did you decide not to attend your appointment with the national retinopathy screening programme? | Already have a full eye screen with another HCP?  Couldn’t travel to appointment?  Don’t have any symptoms?  Newly diagnosed with diabetes? |
| **To finish**  Is there anything you would change about the process? Why?  Is there anything else you want to mention about your experience? |  |

**HEALTH PROFESSIONAL TOPIC GUIDE**

This is part of a larger topic guide developed to explore the implementation of the National Clinical Care Programme for Diabetes.^[[1]](#footnote-1)^ Only questions relating to diabetic retinopathy screening are presented here.

| **Topic** | **Prompt/probe** |
| --- | --- |
| How is the RetinaScreen programme working in this area?  And in your opinion how does RetinaScreen compare to the service that was in this area before?  **Consequences of de-implementation**  RetinaScreen have reported that the national uptake of the screening programme is approximately 50%*, which is below the ideal level. It has been suggested that this is actually lower than what was achieved with previous screening models in certain areas (i.e. there has been a drop off in patient attendance). Why do you think that is?  Have your patients asked you about the RetinaScreen programme?  **Flagging RetinaScreen to patients**  In areas where there was a pre-existing screening service it has been suggested that some endocrinologists weren’t as willing to engage with the new service and refer patients to the RetinaScreen. Why do you think that is?  **Registering patients with RetinaScreen**  In previous interviews it has been suggested that the DRSP register is incomplete. Why do you think that is? | Better- why? Not as good-why?  Patient prefers existing/previous service, why?  -Familiarity or convenience of previous screening location/screening provider  -GP/nurse flags RetinaScreen to patients? Why? Why not?  -Optometrists/ ophthalmologists may not want to lose their patients, why?  -Fit for purpose service already provided  -No clinical need- patient already receiving retinal screening at another service  -ongoing issues between the ICGP and the HSE  -GPs not reimbursed for extra work  -the way in which the RetinaScreen has set-up registration process (i.e. patients can’t register themselves)  - GP already overburdened with patients  - Why are some GP’s registering their patients? |

*At the time of the interview this was the most recent estimate

1. McHugh S, Tracey ML, Riordan F, et al. Evaluating the implementation of a national clinical programme for diabetes to standardise and improve services: a realist evaluation protocol. *Implementation Science*. 2016;11(1):107. [↑](#footnote-ref-1)
