# Supplementary material for: Development of an intervention to facilitate implementation and uptake of diabetic retinopathy screening
Source: Implement Sci. 2020 May 19;15:34. doi: 10.1186/s13012-020-00982-4 (PMC7236930; doi:10.1186/s13012-020-00982-4)
Supplement: Supplementary file 2 — Additional file 2 Table S1. Theoretical Domains Framework: definitions and coding structure developed by Graham-Rowe et al. [26]. [file 13012_2020_982_MOESM2_ESM.docx]

| **Suppl. Table 1 Theoretical Domains Framework: definitions and coding structure developed by Graham-Rowe et al. [27].** | |
| --- | --- |
| **TDF domain and definition** | **Interpretation as it relates to diabetic retinopathy** |
| Knowledge: awareness of the existence of something | In the context of this study, knowledge of the condition/scientific rationale could relate to the patient‘s knowledge of:  diabetes, diabetic retinopathy and the link between the two.   - knowledge about rationale for screening and frequency of screening. - knowledge of the procedure and potential treatments.   Knowledge may be both correct and incorrect but must relate/link to attendance |
| Skills: ability or proficiency acquired through practice | In the context of this study, skills/competence of the patient may include:  diabetes self-management training and education (including the DRS procedure and importance of regular attendance)  Skills may be both present and absent |
| Social professional role and identity: a coherent set of behaviours and displayed personal qualities of an individual in a social or work setting | In the context of this study, professional role may relate to the extent that healthcare professionals feel that providing diabetic retinopathy: screening; education; prompts to attend; recommendations; appointment setting is part of their professional role Personal identity may relate to how a patient identifies with:   - their illness (diabetes) - their view of a typical person who attends/does not attend screening |
| Beliefs about capabilities: acceptance of the truth/reality about or validity of an ability, talent or facility that a person can put to constructive use | In the context of this study, beliefs about capabilities relates to patients‘ judgments on their ability to attend screening including beliefs about their:   - physical/mental ability or confidence to make a screening appointment and/or attend a screening appointments |
| Optimism: confidence that things will happen for the best or that desired goals will be attained | In the context of this study, optimism related to a patient‘s judgment regarding:   - their susceptibility to diabetes related problems, specifically diabetic retinopathy   This includes: Optimism, pessimism, unrealistic optimism. |
| Beliefs about consequences: acceptance of the truth/reality about or validity of outcomes of a behaviour in a given situation | In the context of this study, beliefs about consequences relates to patients‘ judgments on:   - the purpose, value, and effectiveness of screening - negative/positive outcomes of screening |
| Reinforcement: increasing the probability of a response by arranging a dependent relationship, or contingency, between the response and a given stimulus | In the context of this study, reinforcements relate to patients‘ judgments on:   - receiving a reward/incentive for attend screening - receiving a punishment if they do not attend screening |
| Intentions: conscious decision to perform a behaviour or a resolve to act in a certain way | In the context of this study, intentions relate to patients‘ statements on:   - their intention to attend/not to attend screening - their intention to continue to/stop attending screening |
| Goals: mental representation of outcomes or end states that an individual wants to achieve | In the context of this study, goals relate to patients‘ statements on:   - the goals they wish to achieve from attending screening (e.g. preserve/protect vision) - competing goals (goals that might conflict with screening attendance) |
| Memory attention decision processes: ability to retain information, focus selectively on aspects of the environment and choose between two or more alternatives | In the context of this study, memory, attention and decision processes relate to patients‘ statements on:   - patients’ ability to remember to make/attended a screening appointment - how they decide whether to attend or not - feeling overwhelmed with diabetes/multiple appointments or other life circumstances. |
| Environmental context and resources: any circumstances of a person‘s situation or environment that discourages or encourages the development of skills and abilities,  and adaptive behaviour indep | In the context of this study, environmental context and resources relates to patients‘ perceptions of the:   - Time they have to attend - Financial resources they have to attend - Accessibility of the screening service - Resources available within the screening service, hospital, clinic.   This can include the absence or presence of resources |
| Social influences: interpersonal processes that can cause an individual to change their thoughts, feeling or behaviours. | In the context of this study, social influences relate to patients‘ statements expressing the influence of others on attending screening. Including:   - presence/absence of support from friends/family - trust of/respect in related HCPs or belief in their authority - past experiences with HCPs - community groups/wider society |
| Emotion: a complex reaction pattern, involving experiential, behavioural and physiological elements, by which the individual attempts to deal with a personally significant matter or event | In the context of this study, emotions relate to patients‘ statements of expressing their emotional reaction/state relating to:   - attending a screening attendance  a potential diagnosis following a screening appointment - loss of vision/sight   This could also include expression or emotions of the HCP |
| Behavioural regulation: anything aimed at managing or changing objectively observed or measured actions | In the context of this study, behavioural regulation relates to the patients‘ or HCPs‘ statements about steps taken to provide or use:   - techniques/processes to remember/remind patients to attend screening - techniques/processes to ensure patients attend screening |
