# Supplementary material for: Development of an intervention to facilitate implementation and uptake of diabetic retinopathy screening
Source: Implement Sci. 2020 May 19;15:34. doi: 10.1186/s13012-020-00982-4 (PMC7236930; doi:10.1186/s13012-020-00982-4)
Supplement: Supplementary file 3 — Additional file 3. Questionnaire. [file 13012_2020_982_MOESM3_ESM.docx]

**Questionnaire**

Here is a list of ways to improve diabetes eye screening attendance. We want your opinion on whether these things are **acceptable** and **feasible**. When we meet in person we will talk about who is the best person to deliver some of these messages, and when and how they should be delivered (e.g. in person, using a leaflet, a letter or a text message).

For each statement, please circle one number in the **acceptable** category and one in the **feasible** category**.**

**Each number represents the following:**

**1 = Completely disagree**

**2 = Disagree**

**3 = Neither disagree or agree**

**4 = Agree**

**5 = Completely agree**

**For example:**

| Statement | This is acceptable  (you like it, and you think it makes sense) | This is feasible  (you think it can be done) |
| --- | --- | --- |
| Prompt practices to register patients | 1 2 3 4 5 | 1 2 3 4 5 |

| 1. Ways to encourage the person to attend diabetes eye screening | | | | | | | | | | |
| --- | --- | --- | --- | --- | --- | --- | --- | --- | --- | --- |
| Statement | **This is acceptable**  (you like it, it makes sense) | | | | | **This is feasible**  (you think it can be done) | | | | |
|  |  |  |  |  |  |  |  |  |  |  |
| Provide a personal story from someone else with diabetes who… | | | | | | | | | | |
| - is a similar age and profile to them and explains how screening was a way for them to take charge of their health. | 1 2 3 4 5 | | | | | 1 2 3 4 5 | | | | |
| - has retinopathy and tells them about the benefits of screening (e.g. reassured all is ok, treatment stops things getting worse) | 1 2 3 4 5 | | | | | 1 2 3 4 5 | | | | |
| - has retinopathy and tells them it is important to go to screening before it is too late, there may be no symptoms and everyone with diabetes is at risk. | 1 2 3 4 5 | | | | | 1 2 3 4 5 | | | | |
| - wishes they went to screening sooner who prompts the person to think about the regret they will feel if they do not attend screening. | 1 2 3 4 5 | | | | | 1 2 3 4 5 | | | | |
| - explains there is no harm from drops used during screening and the overall benefits outweigh the short-term discomfort. | 1 2 3 4 5 | | | | | 1 2 3 4 5 | | | | |
| - provides an observable example that shows them how to consent or attend. | 1 2 3 4 5 | | | | | 1 2 3 4 5 | | | | |
| - delivers a message recognising the anxiety people might feel but emphasizes the positive consequences of attending. | 1 2 3 4 5 | | | | | 1 2 3 4 5 | | | | |
| - prompts the person to imagine the outcomes of attending vs. not attending (knowing all is ok, treatment available vs. not knowing, they could have eye damage). | 1 2 3 4 5 | | | | | 1 2 3 4 5 | | | | |
|  | **This is acceptable**  (you like it, and think it makes sense) | | | | | **This is feasible**  (you think it can be done) | | | | |
| Someone in the practice could… | | | | | | | | | | |
| - encourage the person to attend screening | 1 2 3 4 5 | | | | | 1 2 3 4 5 | | | | |
| - tell the person that they approve of screening and hope the person will attend. | 1 2 3 4 5 | | | | | 1 2 3 4 5 | | | | |
| - persuade the person they will be able to attend screening (e.g. help them to think about times they successfully managed their diabetes or attended appointments) | 1 2 3 4 5 | | | | | 1 2 3 4 5 | | | | |
| - send or give a take-home a reminder to the person to consent and attend their screening appointment. | 1 2 3 4 5 | | | | | 1 2 3 4 5 | | | | |
| - explain the difference between routine eye checks and the screening test, what both tests can and cannot tell them, and that routine checks are not a substitute. | 1 2 3 4 5 | | | | | 1 2 3 4 5 | | | | |
| - explains there is no harm from drops used during screening and the overall benefits outweigh the short-term discomfort. | 1 2 3 4 5 | | | | | 1 2 3 4 5 | | | | |
| - advise the person how to consent to screening and to ask for help if they are unable/unsure about how to do this. | 1 2 3 4 5 | | | | | 1 2 3 4 5 | | | | |
| - tell the person that after their appointment they will be reassured or they can get treated in time to stop things getting worse. | 1 2 3 4 5 | | | | | 1 2 3 4 5 | | | | |
| - explain how it’s important to go to screening before it is too late, they personally are at risk and that screening applies to them. | 1 2 3 4 5 | | | | | 1 2 3 4 5 | | | | |
| - encourage the person to think of screening not as something extra, but as part of the whole package of self-management. | 1 2 3 4 5 | | | | | 1 2 3 4 5 | | | | |
| - help the person to make a plan about when and where they will consent and how they will attend when they get their appointment. | 1 2 3 4 5 | | | | | 1 2 3 4 5 | | | | |
| Other ideas to encourage the person with diabetes to consent or attend | | | | | | | | | | |
| - arrange for support from family/friends (e.g. encouragement to consent/attend). | 1 2 3 4 5 | | | | | 1 2 3 4 5 | | | | |
| - advise/arrange for practical support from family/friends (e.g. identify transportation). | 1 2 3 4 5 | | | | | 1 2 3 4 5 | | | | |
| - provide a message from the screening service about why they want the person to attend (e.g. our priority is to preserve your vision) and a reminder the service is free. | 1 2 3 4 5 | | | | | 1 2 3 4 5 | | | | |
| - draw the person’s attention to the number of people like them who have attended. | 1 2 3 4 5 | | | | | 1 2 3 4 5 | | | | |
| - the person ticks off a checklist when they have consented/attended. | 1 2 3 4 5 | | | | | 1 2 3 4 5 | | | | |

| 1. Ways that encourage the practice staff to make sure person attends | | |
| --- | --- | --- |
| Statement | **This is acceptable**  (you like it, and think it makes sense) | **This is feasible**  (you think it can be done) |
| - provide practice with observable example/information on how to check and register people with diabetes. | 1 2 3 4 5 | 1 2 3 4 5 |
| - prompt practice to check the register during consultation and register person if necessary (e.g. electronic reminder) | 1 2 3 4 5 | 1 2 3 4 5 |
| - prompt practice to encourage the person to consent/attend & provide information on the benefits | 1 2 3 4 5 | 1 2 3 4 5 |
| - provide a new resource to the practice (e.g. researcher checks if person registered, consented and/or attended) | 1 2 3 4 5 | 1 2 3 4 5 |
| - provide checklist of ways to encourage consent/attendance | 1 2 3 4 5 | 1 2 3 4 5 |
| - establish a way for the practice to monitor and record their efforts to promote attendance | 1 2 3 4 5 | 1 2 3 4 5 |
| - identify someone in the practice to help the person to register and consent. | 1 2 3 4 5 | 1 2 3 4 5 |
| Tell practices about… | | |
| - the benefits to the practice when their patients attend (e.g. receiving timely results, they have access to local service) | 1 2 3 4 5 | 1 2 3 4 5 |
| - consequences when their patients do not attend (e.g. eye damage, costs of missed appointments). | 1 2 3 4 5 | 1 2 3 4 5 |
| Use a personal story from a patient to tell practices… | | |
| - the benefits and risks to patients of attending/not attending | 1 2 3 4 5 | 1 2 3 4 5 |
| - patients are more likely to attend screening if a health professional prompts or encourages them to do so. | 1 2 3 4 5 | 1 2 3 4 5 |
| Give practices feedback on… | | |
| - number of their patients who have not registered, consented or attended | 1 2 3 4 5 | 1. 2 3 4 5 |
| - the differences between % attending from their practice and other practices | 1 2 3 4 5 | 1 2 3 4 5 |
| - national or international uptake or targets | 1 2 3 4 5 | 1 2 3 4 5 |
| - use a trusted source to deliver feedback and messages (e.g. colleague) | 1 2 3 4 5 | 1 2 3 4 5 |

**Please write comments or suggestions here about the ideas listed above:**
