# Supplementary material for: Development of an intervention to facilitate implementation and uptake of diabetic retinopathy screening
Source: Implement Sci. 2020 May 19;15:34. doi: 10.1186/s13012-020-00982-4 (PMC7236930; doi:10.1186/s13012-020-00982-4)
Supplement: Supplementary file 4 — Additional file 4. PubMed search strategy. [file 13012_2020_982_MOESM4_ESM.docx]

**PubMed search strategy**

| **Search #** | **Terms (title or abstract)** |
| --- | --- |
| 1 | improv* OR increase* |
| 2 | screening |
| 3 | uptake OR attendance |
| 4 | reminder* |
| **5** | **4 AND 3 AND 2 AND 1** |
| **6** | **(text messag* OR telephone OR “phone call” OR “phone calls” OR “mailed letter” OR “mailed letters” OR leaflet*)** |
| 7 | 6 AND 1 |
| 8 | 6 AND 2 |
| 9 | 6 AND 3 |
| **10** | **(patient story OR patient stories OR patient testimonial* OR patient narrative*)** |
| 11 | 10 AND 1 |
| 12 | 10 AND 2 |
| 13 | 10 AND 3 |
| 14 | endorse* |
| 15 | 14 AND 1 |
| 16 | 14 AND 2 |
| 17 | 14 AND 3 |
| **18** | **“GP endorsement”** |
