# Supplementary material for: Development of an intervention to facilitate implementation and uptake of diabetic retinopathy screening
Source: Implement Sci. 2020 May 19;15:34. doi: 10.1186/s13012-020-00982-4 (PMC7236930; doi:10.1186/s13012-020-00982-4)
Supplement: Supplementary file 5 — Additional file 5: Table S2. Patient characteristics. [file 13012_2020_982_MOESM5_ESM.docx]

| **Suppl. Table 1 Patient characteristics (n = 47)** |  |  |
| --- | --- | --- |
| **Characteristic** | **N** | **%** |
| **Gender*** |  |  |
| Male | 17 | 36 |
| Female | 10 | 21 |
| Missing | 20 | 43 |
| **Age group (years)** |  |  |
| 18-39 | 6 | 13 |
| 40-64 | 21 | 45 |
| 65+ | 9 | 19 |
| Missing | 10 | 21 |
| **Type of diabetes** |  |  |
| Type 1 | 16 | 34 |
| Type 2 | 27 | 57 |
| Missing | 4 | 9 |
| **Duration of diabetes diagnosis** |  |  |
| 1-4 years | 9 | 19 |
| 5-9 years | 8 | 17 |
| 10+ years | 11 | 23 |
| Missing | 19 | 40 |
| **Healthcare cover** |  |  |
| Medical card | 20 | 43 |
| Private | 20 | 43 |
| Missing | 7 | 15 |
