# Supplementary material for: Development of an intervention to facilitate implementation and uptake of diabetic retinopathy screening
Source: Implement Sci. 2020 May 19;15:34. doi: 10.1186/s13012-020-00982-4 (PMC7236930; doi:10.1186/s13012-020-00982-4)
Supplement: Supplementary file 6 — Additional file 6: Table S3. a. Modifiable patient-level barriers and enablers and corresponding TDF mapped to BCTs. b. Modifiable professional-level barriers and enablers and corresponding TDF mapped to BCTs based on published expert consensus about effectiveness for behaviour change [5, 6] and the mapping matrix constructed by Lawrenson et al. [7]. b Modifiable professional-level barriers and enablers and corresponding TDF mapped to BCTs based on published expert consensus about effectiveness for behaviour change (1, 2) and the mapping matrix constructed by Lawrenson et al HTA. (3) a Modifiable patient-level barriers and enablers and corresponding TDF mapped to BCTs based on published expert consensus about effectiveness for behaviour chang e[5, 6] and the mapping matrix constructed by Lawrenson et al. [7]. b Modifiable professional-level barriers and enablers and corresponding TDF mapped to BCTs based on published expert consensus about effectiveness for behaviour change (1, 2) and the mapping matrix constructed by Lawrenson et al. HTA (3). [file 13012_2020_982_MOESM6_ESM.docx]

| **Suppl. Table 3a Modifiable patient-level barriers and enablers and corresponding TDF mapped to BCTs based on published expert consensus about effectiveness for behaviour change[5, 6] and the mapping matrix constructed by Lawrenson et al.[7]** | | | | |
| --- | --- | --- | --- | --- |
| **DOMAIN**  **Barrier (-) or enabler (+)** | **TDF** | **BCTs (long list) ✓ retained** | | **BCTs (short list)** |
| Register is incomplete so patients do not get letter from programme (-) | Environmental Context | - Restructuring the social environment - Restructuring the physical environment - Discriminant (learned) cue - Prompts/ Cues - Avoidance/ changing exposure to cues for the behaviour | - Restructuring the social environment¶^║^ - Prompts/ Cues | |
| Patient cannot self-register; need HCP involvement in this process (-) | Environmental Context |  |  | |
| Programme sends reminders (+) | Environmental Context |  |  | |
| Trust in HCP (-+)^[[1]](#footnote-1)^ | Social influences | - Social support (unspecified) - Social support (emotional) - Social support (practical) - Social reward - Information about others’ approval - **Vicarious consequences/reinforcement** - Restructuring the social environment - **Identification of self as a role model** - Modelling/demonstration by others - **Social comparison** | - Social support (unspecified)¶^║^ - Social support (emotional)^║^ | |
| HCP recommends they attend screening (+) | Social influences |  | - Social reward¶ - Social support (practical)¶^║^ | |
| HCP explains importance of screening (+) | Social influences |  | - Information about others’ approval^║^ | |
| HCP actions to support patient attendance: register patients (+), check registration (+), facilitate process of consenting to the programme (+) | Social influences |  | - Restructuring the social environment¶^║^ - Modelling/demonstration by others¶^║^ | |
|  | Social influences |  |  |  |
| Support/recommendation from friends/family members (+) | Social influences |  |  |  |
| Forgetting to consent or attend (-) | Memory, attention, decision processes | - Prompts/cues - Action planning - Self-monitoring of behaviour - **Self-monitoring of outcome of behaviour** | - Prompts/cues¶* - Action planning¶^║^ - Self-monitoring of behaviour¶^║^ | |
| Knowing it’s a routine part of their care (+) | Memory, attention, decision processes |  |  |  |
| Being checked elsewhere – no need (-) | Memory, attention, decision processes |  |  |  |
| If patients do not have symptoms they feel they do not need to attend screening. Some patients do not link symptoms to their diabetes. (-) | Memory, attention, decision processes |  |  | |
| Confusion between new screening and existing, routine eye tests (-) | Knowledge | - Information about health consequences - Information about social and environmental consequences - **Information about emotional consequences** - **Biofeedback** - Antecedents (Instruction on how to perform the behaviour; Reattribution; **Behavioural experiments**) - **Feedback on behaviour** - Salience of consequences | - Information about health consequences¶^║^ - Salience of consequences¶^║^ | |
|  | Knowledge |  |  |  |
| Lack of awareness of illness (link between diabetes and eye damage) (-) | Knowledge |  |  | |
|  |  |  |  | |
| Misunderstanding treatment options i.e. there is no treatment for eye damage so no point attending to find out (-)  Believe service is ‘looking for money’ (-)  Understanding it is a ‘free service’ (+). | Knowledge  Knowledge |  |  | |
| [**HCP] Beliefs about patient physical capabilities** |  |  |  | |
| Patient finds it difficult to consent via phone process (-) | Skills | - Instruction on how to perform the behaviour - Modelling/demonstration by others | - Instruction on how to perform the behaviour¶^║^ - Modelling/demonstration by others¶^║^ | |
| [**Patient] Beliefs about mental capabilities** |  |  |  | |
| Patients are disengaged with diabetes in general or ‘in a rut’ (-) | Beliefs about capabilities | - Focus on past success - Verbal persuasion to boost self-efficacy | - Focus on past success¶^║^ - Verbal persuasion to boost self-efficacy¶ | |
| Patient is already in routine of going for tests (+) | Beliefs about capabilities |  |  | |
|  |  | - Information about emotional consequences - Information about social and environmental consequences - Salience of consequences - **Covert sensitization** - **Self-monitoring of behaviour** - **Self-monitoring of outcome of behaviour** - Information about health consequences - Anticipated regret - Pros and cons - **Vicarious reinforcement** - **Threat** - Comparative imagining of future outcome - **Feedback on behaviour** - Feedback on outcomes of behaviour - **Biofeedback** - Persuasive communication (credible source) | - Information about social and environmental consequences¶ | |
|  |  |  | - Persuasive communication (credible source)¶* | |
| Perceived necessity of screening (-) | Beliefs about consequences |  | - Salience of consequences¶^║^ | |
| Screening provides valuable information on eye health status (-+)   - Early detection (+) - Reassurance (+) | Beliefs about consequences |  | - Anticipated regret^║^ - Comparative imagining of future outcome - Pros and cons^║^ - Information about emotional consequences¶ | |
| Salient consequences; experiencing complications or knowing others who have (+) | Beliefs about consequences |  |  | |
| Harmful effect of the screening procedure (-) | Beliefs about consequences |  |  | |
| Anticipated negative outcome (-)   - Fear of a bad result (-) | Beliefs about consequences |  | - Feedback on outcomes of behaviour¶* | |
| Lack of awareness of the importance of screening (-) | Beliefs about consequences |  |  | |
| Fear or anxiety about vision loss (+) | Emotion | - Reduce negative emotions - Information about emotional consequences - **Conserving mental resources** - **Self-assessment of affective consequences** - Social support (emotional) | - Reduce negative emotions - Social support (emotional) - Information about emotional consequences¶ | |
| Confident not at risk (-) | Emotion |  |  | |
| Ownership or responsibility over their illness (+) | Social professional role | - None |  | |
| Prioritising health (eye health, staying healthy, better quality of life) (+) | Goals | - Action Planning - Goal Setting (Behaviour) - **Goal Setting (Outcome)** - Review behaviour goals - **Review of Outcome Goal(s)** | - Action Planning¶^║^ - Goal Setting (Behaviour)¶* - Review behaviour goals¶^║^ | |
| *****part of effective interventions to improve attendance  ¶already operationalised as part of existing interventions to improve attendance  ^║^Other evidence of effectiveness of the BCT from the wider literature  No evidence but makes sense to use this BCT to target specific barrier or enabler  **Does not make sense for this barrier or enabler although mapped to the corresponding domain** | | | | |

| **Suppl. Table 3b Modifiable professional-level barriers and enablers and corresponding TDF mapped to BCTs based on published expert consensus about effectiveness for behaviour change^(1, 2)^ and the mapping matrix constructed by Lawrenson et al HTA.^(3)^** | | | |
| --- | --- | --- | --- |
| **DOMAIN**  **Barrier (-) or enabler (+)** | **TDF** | **BCTs (long list)** | **BCTs (short list)** |
| **OPPORTUNITY** |  |  |  |
| Length of time taken to register patients or to check register (-) means that patient registration is impeded or supported by availability of practice resources (-+)  HCPs reported there was ‘no money’ in tracking or encouraging patients who have not attended (-) | Environmental context | - **Restructuring the physical environment - adding objects to the environment** - Restructuring the social environment - **Discriminative (learned) cue** - **Avoidance/changing exposure to cues for behaviour** - **Prompts/cues** | - Restructuring the social environment¶* |
| HCP who have attended a diabetes course know that registering and ensuring patients attend screening is part of their care (+) | Knowledge | - **Information about health consequences** - **Information about social and environmental consequences** - **Information about emotional consequences** - **Biofeedback** - **Antecedents** (**Instruction on how to perform the behaviour**; **Reattribution; Behavioural experiments**) - Feedback on behaviour - **Salience of consequences** | - Feedback on behaviour^║^ |
| Lacking knowledge on service uptake / DNAs in their area or practice (-) | Knowledge |  |  |
| HCP just accept that registering and ensuring patients attend screening is part of routine care (+) | Memory, attention, decision processes | - Prompts/cues - **Action planning** - **Self-monitoring of behaviour** - **Self-monitoring of outcome of behaviour** | - Prompts/cues¶ |
| HCP feel that once patient is attending screening programme it is facilitates follow-up care with patients e.g. they know the patient status re: eye health, they get letters back so they can keep track (+) | Beliefs about consequences | - Persuasive communication (credible source) - **Information about emotional consequences** - Information about social and environmental consequences - **Salience of consequences** - **Covert sensitization** - **Self-monitoring of behaviour** - **Self-monitoring of outcome of behaviour** - **Information about health consequences** - **Anticipated regret** - **Pros and cons** - **Vicarious reinforcement** - **Threat** - **Comparative imagining of future outcome** - **Feedback on behaviour** - Feedback on outcomes of behaviour - **Biofeedback** | - Persuasive communication (credible source)¶* - Feedback on outcomes of behaviour¶ - Information about social and environmental consequences¶ |
| Recognise that attending national screening programme will mean local access for patients (+) | Beliefs about consequences |  |  |
| HCP see it as part of their role to register patients (+) | Social professional role | - None |  |
| Passionate about role and delivering high-quality diabetes care (+) | Social professional role |  |  |

1. Patients who trust doctor will do what they say (and attend) (+); Patients who trust existing (familiar) eye care provider means they do not attend new screening service (-) [↑](#footnote-ref-1)
