# Supplementary material for: Development of an intervention to facilitate implementation and uptake of diabetic retinopathy screening
Source: Implement Sci. 2020 May 19;15:34. doi: 10.1186/s13012-020-00982-4 (PMC7236930; doi:10.1186/s13012-020-00982-4)
Supplement: Supplementary file 7 — Additional file 7: Table S4. Organisational factors identified from health professional and patient interviews, coded according to CFIR and TDF. [file 13012_2020_982_MOESM7_ESM.docx]

| **Suppl. Table 4 Organisational factors identified from health professional and patient interviews, coded according to CFIR and TDF** | | | |
| --- | --- | --- | --- |
| **CFIR Construct** | **TDF domain** | **Sub-theme** | **Implications for intervention components** |
| **Outer context** |  |  |  |
| **External policies and incentives** | Environmental context | HCPs reported there was ‘no money’ in tracking or encouraging patients who have not attended. Since the introduction a new financial incentive scheme, the cycle of care*, they felt there was some resourcing in place for this work for GMS patients. | - Provide practices with reimbursement and communicate this in recruitment materials |
|  | Environmental context | Accessibility of screening clinics influenced patient attendance; i.e., if a centre was close by, they were inclined to go, if it was farther away they were unable to attend if they did not have transport (e.g. friends/family, public transport) to and from the appointment. | - Non-modifiable factor may moderate intervention fit. Include in process evaluation (patient interviews). Practices will record why patients did not attend as part of the phone call. |
|  | Environmental context | The flexibility of the service, the ability to ring the programme and make a new, more suitable appointment helped people to attend. | - Select messages to include in the information materials which highlight it is possible to reschedule their appointment to a time which suits them best. |
|  | Environmental context | People cannot attend because of competing demands, including being unable to take time off work or having family dependents and other commitments. | - Select messages to include in the information materials which highlight it is possible to reschedule their appointment to a time which suits them best. |
|  | Environmental context | The service is free, and people attend if they are aware of this. | - Select messages to include in the information materials which highlight the service is free. |
| **Inner setting** |  |  |  |
| **Readiness for implementation** |  |  |  |
| Access to information and knowledge | Knowledge  Knowledge  Knowledge | HCPs who have attended courses (e.g. diabetes module) or a talk by a screening programme representative are aware of RetinaScreen and know how important it is.  HCPs lack knowledge on service uptake in their region; having this knowledge could mean they know who to target.  HCPs are confused about whether patients who are seen elsewhere (e.g. hospital service) also need to attend screening with the national programme; often they will err on the side of caution and advise patients to go to both. | - Select messages to include in brief training which reiterate the purpose and value of screening and the importance of their role as a strong influence on patient. - Practices conduct audit to determine non-attenders. - As part of the brief training clarify this with practice staff they should encourage patients to attend screening with the national programme even the patient has been seen elsewhere. |
| Available resources | Environmental context | One HCP had an administrative resource to register patients for RetinaScreen so it was not an issue. | - Non-modifiable factor. Include in process evaluation. Record who practices assign this task to. Allow some flexibility in terms of which staff member undertakes the practice audit and delivers the reminder messages |
| Compatibility | Environmental context  Social professional role | Some HCPs work at practices where there are already structured reviews in place (and this prompts them to remind patients about screening). For example, they may have been part of a long-standing diabetes initiative or have started cycle of care reviews.  HCPs follow up and remind patients to attend because they believe that it is just ‘part of diabetes care’ and part of what they do. | - Non-modifiable factor which may moderate intervention fit. Include in process evaluation (i.e. record baseline practice set-up, for example, whether they are new to structured reviews) - Select messages to include in brief training which reiterate the purpose and value of screening and the importance of their role as a strong influence on patient. |
|  | Beliefs about consequences | Patients dislike the drops administered during appointments. They are worried about them (i.e. stinging, harm to eyes) or find them inconvenient as they cannot see properly for a period afterwards. | - Select messages to include in the information materials which emphasise the effects of the drops are short term vs. the benefits of screening. |
| Culture | Social professional role | HCPs follow up and remind patients to attend because there is a belief at a practice-level in providing good diabetes care and/or they personally are passionate and proactive about their role delivering diabetes care. | - Select messages to include in brief training which highlight the purpose and value of the intervention. |
| **Intervention characteristics** |  |  |  |
| Complexity | Environmental context  Environmental context | The process of checking the register/registering patients (online or via phone) is lengthy.  HCPs felt this process was too resource intensive. | - Provide practices with reimbursement to encourage them to prioritise registration. Communicate details of reimbursement in recruitment materials. - Reimburse practices for the time taken to carry out an audit of their patients with diabetes. |
| Relative advantage | Beliefs about consequences | Advantages from HCP perspective: if patients are part of the programme, it is easier to track them and manage their follow up (e.g. HCPs know whether they have retinopathy or not and what stage)  Patients enrolled in the programme have local access to screening rather than having to attend hospital. | - Select messages to include in the brief training which highlight these benefits for practices. |

HCP, health care professionals; CFIR, Consolidated Framework for Implementation Research; TDF, Theoretical Domains Framework

*In October 2015, the Diabetes Cycle of Care was introduced. The scheme remunerates GPs to provide two free structured review visits per year to patients with type 2 diabetes with a General Medical Services (GMS) card; the means-tested General Medical Services (GMS) scheme provides medical care to approximately 40% of the population [71]. General practitioner (GP) visits are free for those with a GMS card, as are hospital care and medications (except for a prescription co-payment).
