# Supplementary material for: Development of an intervention to facilitate implementation and uptake of diabetic retinopathy screening
Source: Implement Sci. 2020 May 19;15:34. doi: 10.1186/s13012-020-00982-4 (PMC7236930; doi:10.1186/s13012-020-00982-4)
Supplement: Supplementary file 8 — Additional file 8: Table S5. a Patient-level barriers or enablers mapped to TDF, BCT and operationalisation. b Professional-level barriers or enablers mapped to TDF, BCT and operationalisation. c. Organisational level barriers or enablers mapped to TDF, BCT and operationalisation. [file 13012_2020_982_MOESM8_ESM.docx]

| **Suppl. Table 5 (a) Patient level barriers or enablers mapped to TDF, BCT and operationalisation** | | | | |
| --- | --- | --- | --- | --- |
| **Barrier or enabler** | **TDF** | **Selected BCT** | **Operationalised** | **Excluded (**🗶) **/included (**✓) **component including notes to justify omission** |
| HCP actions support patient attendance: they register patients, check registration, facilitate consent | Environmental context | Restructuring the social environment¶^║^ | Team member (GP or nurse) is in place to assist patient with registration and consent. Make patient aware that they can ask for help from family, friends or HCPs. | ✓ May be more feasible if administrator at the practice has this role. |
| Recommendation by HCP to attend screening encourages patients to attend | Social influences | Social support (unspecified)¶^║^ | GP or nurse provides general encouragement or reassurance to attend appointment. | ✓ |
|  |  | Providing information about others’ approval^║^ | GP or nurse expresses hope that the patient will attend screening. | ✓ |
|  |  | Social reward¶ | Patient is sent congratulatory letter following attendance at their appointment and/or GP or nurse indicates their approval after attendance. | 🗶 Requires change to RetinaScreen processes. |
| Support/recommendation from friends/family members encourages patients to attend | Social influences | Social support (unspecified)¶^║^ | Arranging for support from friends/family e.g. general encouragement or reassurance to attend appointment. | ✓ |
|  |  | Demonstration of the behaviour¶  Persuasive communication (credible source)¶* | Provide an observable example of attending screening appointment e.g. letter or leaflet including a testimonial to model eye screening behaviour. | ✓ |
| Patient finds it difficult to consent via phone process (-) | Skills | Instruction on how to perform the behaviour¶^║^  Social support (practical)¶ | Provide advice to people on how they can arrange an appointment (e.g. letter (reminder)) - if they do not have an appointment, explain how, and specify if they are unable or unsure to ask GP or nurse or friends/family to help them arrange one. | ✓ |
| Patients forget to respond to consent letter and/or forget appointment | Memory, attention, decision processes | Prompts/cues¶* | Remind patient to consent and/or attend their appointment (letter / phone / face to face). | ✓ |
|  |  | Action planning¶^║^  Review behaviour goals¶^║^ | Support patient to develop plan for how often they will attend screening, where it will take place and how they will get their appointment.  HCP prompts patient to review behaviour i.e. whether attended screening or change goals around attendance. | 🗶 Requires extensive input from GP or practice nurse, making strategy more complex and not practical. |
|  |  | Self-monitoring of behaviour¶^║^ | Support patient to tick off a checklist when they have attended screening. | ✓ |
| Patients are confused between screening and routine eye tests and think they do not need to attend the new screening programme.  Patients have been checked elsewhere and decide they do not need to attend. | Memory, attention, decision processes  Knowledge | Information on health consequences¶^║^ | Clarify difference between screening and routine checks, that screening is part of their care; routine checks are not a substitute | ✓ |
| Patients who attend recognise screening as a routine part of their diabetes care. | Knowledge, Memory, attention, decision processes  Goals | Framing/reframing | Encourage patients to adopt a different perspective on retinal screening- not seeing it as something extra but part of the whole package of routine/ optimal self-management | ✓ |
|  |  | Social comparison¶^║^ | Draw patient’s attention to the proportion (or number) of patients at their practice who have attended screening. | ✓ Aware that if numbers low they may not work as intended. May need more specificity as social comparison only works if perceived as a meaningful comparison. |
| If patients do not have symptoms they feel they do not need to attend screening. Some patients do not link symptoms to their diabetes. | Memory, attention, decision processes | Salience of consequences¶^║^  Information about health consequences¶^║^  Anticipated regret^║^ | Convey message that there may be no symptoms, but they personally are at risk and screening applies to them.  Inform patients it is important to go before it’s too late. | ✓  ✓ |
| Patients lack awareness of the link between diabetes and eye damage. | Knowledge | Information about health consequences¶^║^ | Communicate information about risk, i.e. there may be no symptoms, that everyone with diabetes is at risk | ✓ |
| Patients do not attend as they are confident they personally are not at risk. | Emotion | Information about health consequences¶^║^  Persuasive communication (credible source)¶*  Social comparison¶^║^  Anticipated regret^║^ | Communicate information about risk, i.e. there may be no symptoms, that everyone with diabetes is at risk  Use testimonial i.e. previous non-attenders who then developed retinopathy and wished they had gone earlier  Use message to trigger them to think about the degree of regret they will feel if they do not go | ✓  ✓  ✓ |
| Patients who were disengaged with diabetes or ‘in a rut’ feel unable to attend.  Patients who are in a routine of going for tests do not see it as a problem to attend | Beliefs about capabilities | Focus on past success¶^║^  Verbal persuasion to boost self-efficacy¶ | Tell patient they will successfully be able to attend screening appointment (e.g. GP or nurse encourages or reassures patient to attend appointment) – include a message about taking charge of their health.  Help patient to think about last time they attended screening (or another appointment). | ✓  ✓ |
| Patients who attend have an attitude of ownership or responsibility over their diabetes | Professional role and identity | Verbal persuasion to boost self-efficacy¶ | Tell patient they will successfully be able to attend screening appointment (e.g. GP or nurse encourages or reassures patient to attend appointment) – include message about taking charge of their health. | ✓ |
|  |  | Instruction on how to perform the behaviour¶^║^  Demonstration of the behaviour¶  Persuasive communication (credible source)¶*  Social comparison¶^║^ | Provide an observable example of attending screening appointment e.g. letter or leaflet  Include a testimonial to model eye screening behaviour. | ✓  ✓ |
| Patients believe screening service is ‘looking for money’ | Beliefs about consequences | Information on social and environmental consequences  Persuasive communication (credible source)¶* | Screening service communicating positive message about why they want the patient to attend i.e. screening is free and our priority is to preserve your vision | ✓ |
| Patients lack awareness of the importance of screening.  Patients generally do not perceive the necessity of screening.  Patients attend because they believe screening provides valuable information on their eye health status, facilitates early detection of problems and will provide reassurance.  Patients attend because consequences are salient consequences; they have experienced complications or know others who have. | Beliefs about consequences | Information about emotional consequences¶  Information about health consequences¶^║^  Salience of consequences¶^║^  Persuasive communication (credible source)¶* | Provide information on the consequences of attending an appointment; they will be reassured all is ok or can be treated early to stop things getting worse  Include testimonial (e.g. leaflet) from other people with diabetes who suffer from retinopathy and emphasise benefits of attending – early detection and reassurance. | ✓  ✓ |
| Patients do not attend because they anticipate negative outcome of screening and fear a bad result. | Beliefs about consequences | Comparative imagining of future outcome  Pros and cons^║^ | Prompt the patient to imagine and compare likely or possible outcomes following attending vs not attending a screening appointment (e.g. all is ok or can be treated early to stop things getting worse vs not knowing about eye health, could have eye damage).  Advise patient to make list of disadvantages and advantages of attending | **🗶** Other BCTs better target the barriers and enablers. Requires extensive input from GP or practice nurse, making strategy more complex and potentially unfeasible. No evidence for these BCTs as part of strategies to improve screening. |
|  |  | Feedback on outcomes of behaviour¶* (or biofeedback) | After their eye exam, immediately inform patients of the result. | **🗶** Other BCTs better target the barriers and enablers better. Practicality uncertain. |
| Fear or anxiety about vision loss leads patients to attend | Emotion | Reduce negative emotions  Persuasive communication (credible source)¶* | Provide on the consequences of attending an appointment; they will be reassured all is ok or can be treated early to stop things getting worse  Provide information on consequences from credible source (e.g. GP or nurse).  Include testimonial (e.g. leaflet) from other people with diabetes who suffer from retinopathy and emphasise benefits of attending – early detection and reassurance. | ✓  ✓  ✓ |
| Patients are concerned about harmful effect of the screening procedure.  There are negative, short-term effects of screening (e.g. eye drops). | Beliefs about consequences | Persuasive communication (credible source)¶*  Information about health consequences¶^║^ | Provide information from credible source i.e. testimonial from other people with diabetes who have attended - emphasise no harm from drops, temporary discomfort, but not harmful and overall benefits outweigh this | ✓ |
| Patients who attend prioritise their health (eye health, staying healthy, better quality of life). | Goals | Goal Setting (Behaviour)¶* | HCP helps or supports patient to agree on goals for their health as individual diabetes including how often they will attend screening. | **🗶 ‘**Goals’ domain not a priority. Uncertain whether this is feasible and whether it addresses the identified enabler |

*****part of effective interventions to improve attendance at retinopathy screening

¶part of existing interventions to improve attendance at retinopathy screening - ‘workable’

^║^Other evidence of effectiveness of the BCT from the wider literature

Added following review. Note: does not map to TDF but makes sense to apply based on intervention function

| **Suppl. Table 2 (b) Professional-level barriers or enablers mapped to TDF, BCT and operationalisation** | | | | |
| --- | --- | --- | --- | --- |
| **Barrier or enabler** | **TDF** | **Selected BCT** | **Operationalised** | **Excluded (**🗶) **/included (**✓) **component including notes to justify omission** |
| HCP who have attended diabetes courses know that registering and ensuring patients attend screening is part of their care. | 1. Knowledge 2. Memory, attention, decision processes | Prompts/cues¶  Adding objects to environment Restructuring physical environment | Prompt HCPs as part of routine patient consultation to check if patient status (registered / consented / attended) and if not, to assist patient to perform this action. | ✓ |
| HCP register because they just accept that registering and ensuring patients attend screening is part of routine care. |  | Instruction on how to perform the behaviour¶^║^  Demonstration of the behaviour¶ | Provide practice with observable example/info on how to check & register patients | ✓ |
| HCP feel that once patient is attending screening programme it facilitates follow-up care with patients e.g. they know the patient status regarding their eye health; they get letters back from the programme so they can keep track. | Beliefs about consequences | Information about social and environmental consequences¶  Feedback on outcomes of behaviour¶ /Biofeedback | Inform HCP that registering and ensuring patient attends the programme will mean 1) they know their patients’ status 2) patients will be able to get a routine part of their care done closer to home  Information on patient’s status | ✓  **🗶** |
| They recognise that attending national screening programme means more local access for patients. |  | Persuasive communication (credible source)¶* | Use a credible source (peer testimonial, researcher) to inform HCP of above | ✓ |
| HCP register patients and encourage them to attend because they are passionate about their role and believe in delivering high-quality diabetes care. | Social professional role | Persuasive communication (credible source)¶*  Framing/reframing | Using a credible source (peer testimonial, researcher) to…  …encourage HCPs to see influencing patients to attend as part of their role in delivering good diabetes care | ✓  ✓ |

*****part of effective interventions to improve attendance at retinopathy screening

¶part of existing interventions to improve attendance at retinopathy screening - ‘workable’

^║^Other evidence of effectiveness of the BCT from the wider literature

Added following review. Note: does not map to TDF but makes sense to apply based on intervention function

| **Suppl. Table 5 (c) Organisational level barriers or enablers mapped to TDF, BCT and operationalisation** | | | | |
| --- | --- | --- | --- | --- |
| **Barrier or enabler** | **TDF** | **Selected BCT** | **Operationalised** | **Excluded (**🗶) **/included (**✓) **component including notes to justify omission** |
| The (long) length of time taken to register patients or to check the register means that patient registration is impeded or supported by the availability of practice resources.  HCPs reported was ‘no money’ in tracking or encouraging patients who have not attended (-) | Environmental context | Restructuring the social environment¶* | An additional resource is available (e.g. member of research team) to check patient status (registered / consented / attended) and prepare a patient list of same. | 🗶New resource unfeasible. (General Data Protection Regulations 2018 preclude research team member from operating within practices). |
| Lacking knowledge on service uptake / DNAs in their area or practice. | Knowledge | Feedback on behaviour¶^║^  Monitoring of behaviour by others | Give HCP feedback on the % of patient list who have registered /consented / attended; this could include comparator (peer practice, national or international figures) | ✓ Due to the issue outlined above, a member of practice staff will need to conduct an audit to provide this feedback. |
|  |  | Discrepancy between current behaviour and goals’ ¶  ‘Social comparison’¶ | Provide feedback to HCPs on % of patients who have attended screening vs international or peer levels of uptake)  Draw attention to other practice’s performance in terms of patient registration / attendance | ✓  ✓ |
|  |  | Persuasive communication (credible source)¶* | Provide national or international guidelines endorsing the importance of screening | ✓ |
|  |  | Persuasive communication (credible source)¶* | Use a credible source (peer testimonial, researcher) to inform HCP of above | ✓ Due to the issue outlined above, a member of practice staff will need to provide feedback. |

*****part of effective interventions to improve attendance at retinopathy screening

¶part of existing interventions to improve attendance at retinopathy screening - ‘workable’

^║^Other evidence of effectiveness of the BCT from the wider literature

Added following review. Note: does not map to TDF but makes sense to apply based on intervention function.
