# Supplementary material for: Development of an intervention to facilitate implementation and uptake of diabetic retinopathy screening
Source: Implement Sci. 2020 May 19;15:34. doi: 10.1186/s13012-020-00982-4 (PMC7236930; doi:10.1186/s13012-020-00982-4)
Supplement: Supplementary file 9 — Additional file 9: Figure S1. a Ways to encourage patients to attend (practice led). b Ways to encourage patients to attend (narrative led). c Ways to encourage patients to attend (other ideas). d Ways to encourage professionals to prompt patients about screening (feedback). e Ways to encourage professionals to prompt patients about screening (feedback). f Ways to encourage professionals to prompt patients about screening (other ideas). [file 13012_2020_982_MOESM9_ESM.docx]

**
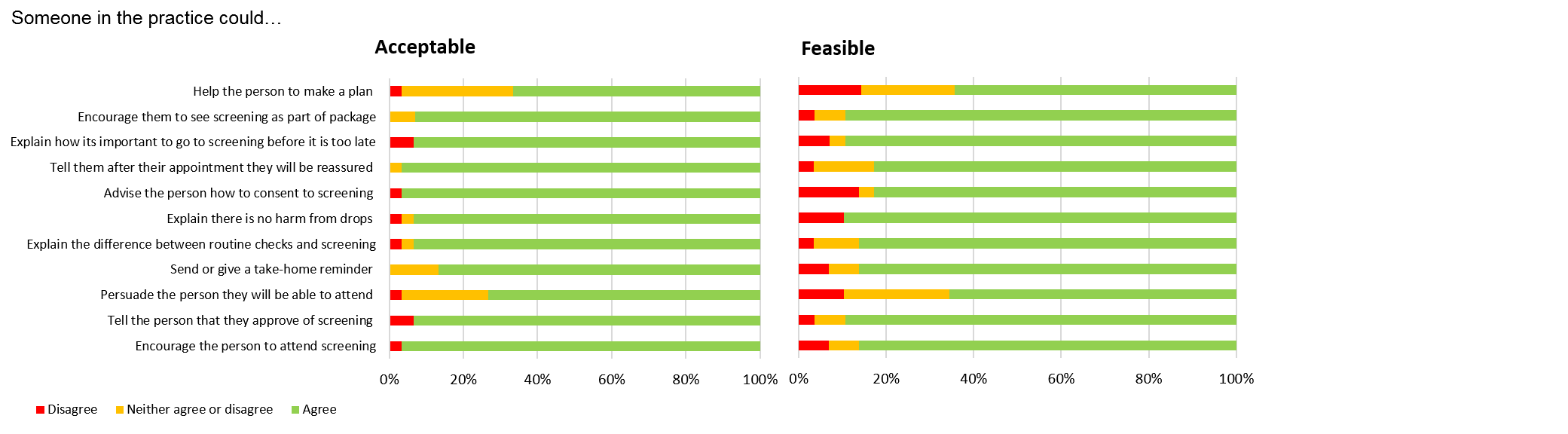
**

Suppl. Figure 1 (a) Ways to encourage patients to attend (practice led)


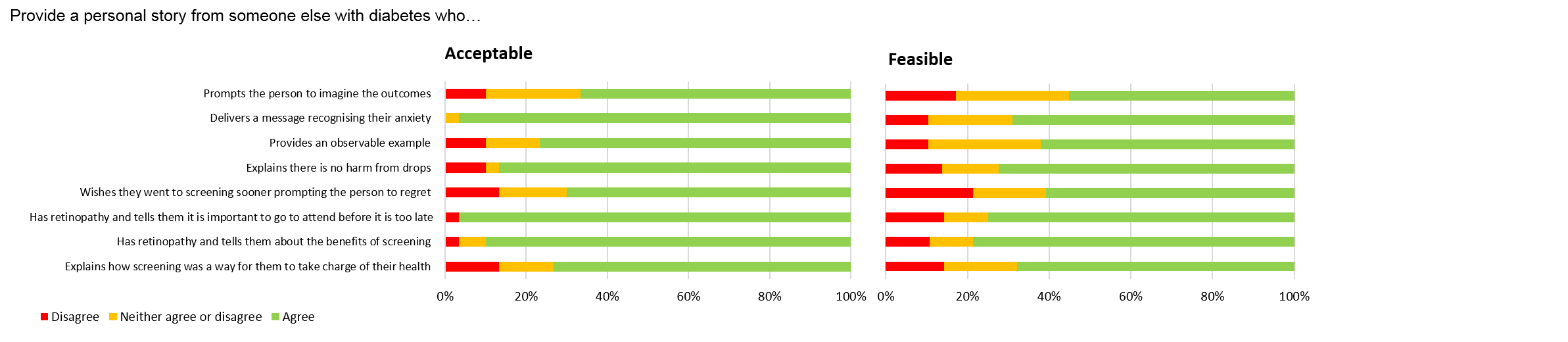


Suppl. Figure 1 (b) Ways to encourage patients to attend (narrative led)


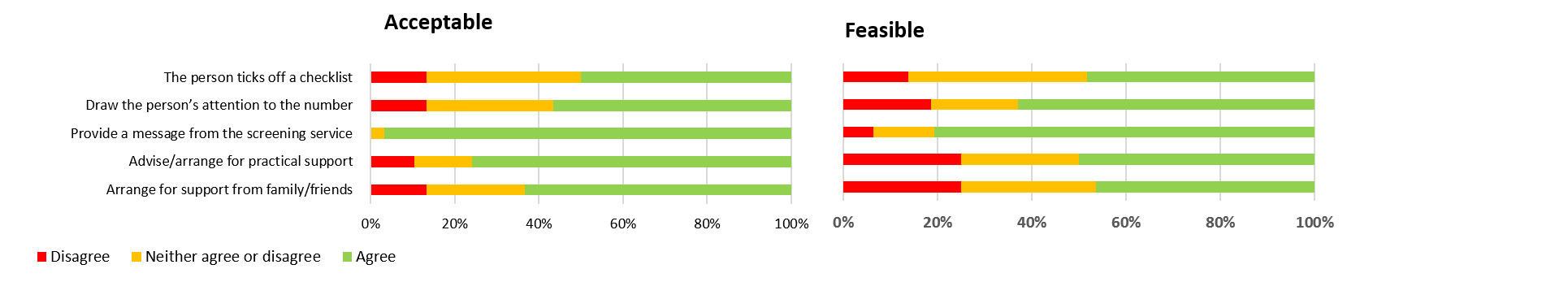


Suppl. Figure 1 (c) Ways to encourage patients to attend (other ideas)


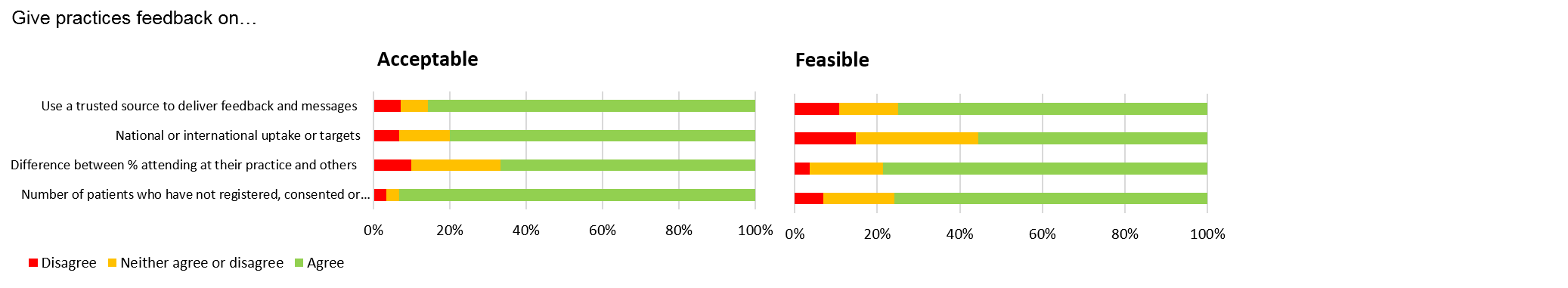


Suppl. Figure 1 (d) Ways to encourage professionals to prompt patients about screening (feedback)


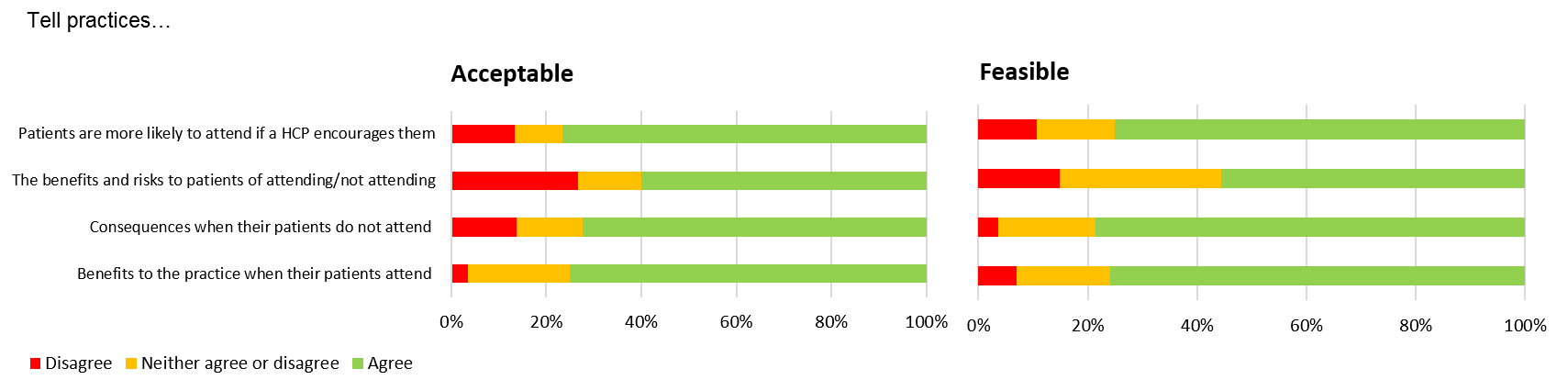


Suppl. Figure 1 (e) Ways to encourage professionals to prompt patients about screening (feedback)


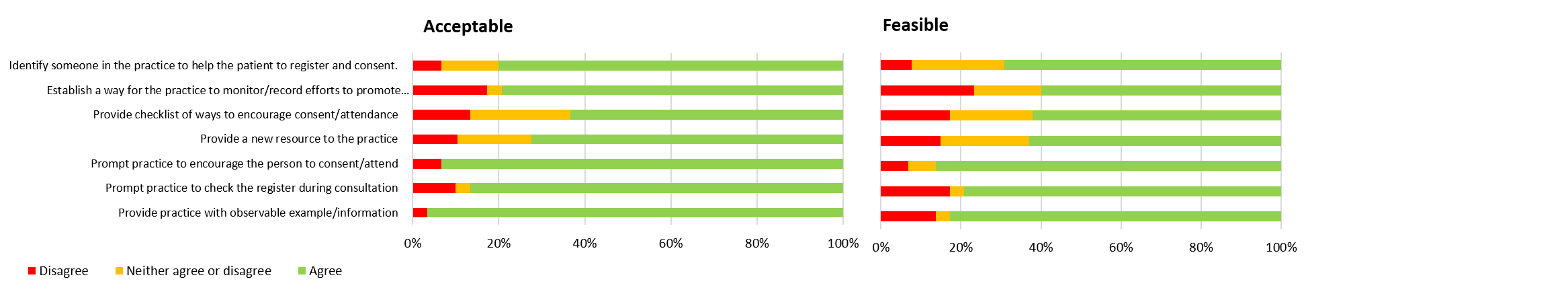


Suppl. Figure 1 (f) Ways to encourage professionals to prompt patients about screening (other ideas)
