# Supplementary material for: Development of an intervention to facilitate implementation and uptake of diabetic retinopathy screening
Source: Implement Sci. 2020 May 19;15:34. doi: 10.1186/s13012-020-00982-4 (PMC7236930; doi:10.1186/s13012-020-00982-4)
Supplement: Supplementary file 10 — Additional file 10. Script. [file 13012_2020_982_MOESM10_ESM.pdf]

1 Our **Records** show that you may not have participated in diabetes eye screening recently with the national RetinaScreen programme.

Do you know about 'Diabetic RetinaScreen'?

RetinaScreen is the national diabetes retinopathy screening programme which offers free, regular retinopathy screening to people with diabetes.

Would you mind if I ask why you haven't participated?

If they are unsure whether they have gone to screening, prompt them re: local screening locations and record the name of the provider who did the screening.

2 We strongly **Recommend** that you participate in screening.

3 Diabetes can cause damage to your eyes. This complication of diabetes is called retinopathy. *Everyone* with diabetes is at **Risk** of developing retinopathy. Even if you have no symptoms, your eyes may already be showing signs of damage. Attending screening dramatically increases the chance of this damage being picked up early and treated in time.

4 After the screening test most people are **Reassured** that their eyes are fine.

<u>If they ask about appointment:</u> It takes about an hour for the appointment. They will add drops to your eyes to temporarily make your pupils larger, and that will temporarily blur your vision slightly. Photographs will be taken of the back of the eyes.

5 The **Routine** eye checks you might have with your optician are different to the screening provided by the national screening service. Even if you are attending an optician you should still participate in the national programme.

6 It's a free service and it's very easy to participate; all you need to do is **Ring** RetinaScreen (1800 45 45 55). If you want help, come in and speak to the nurse. We will send you a reminder letter in a day or two with some more information.
