# Supplementary material for: Development of an intervention to facilitate implementation and uptake of diabetic retinopathy screening
Source: Implement Sci. 2020 May 19;15:34. doi: 10.1186/s13012-020-00982-4 (PMC7236930; doi:10.1186/s13012-020-00982-4)
Supplement: Supplementary file 11 — Additional file 11. Reminder letter. [file 13012_2020_982_MOESM11_ESM.pdf]

HEADED PAPER

<dd> <month> <year>

Dr { }

Address

Important information about diabetes eye screening

Dear { }

Our records show that you may not have participated recently in diabetes eye screening with the national RetinaScreen service. As your GP, I strongly recommend that you participate in screening. *Everyone* with diabetes is at risk of sight loss due to retinopathy. There are about 190,000 people in Ireland with diabetes and 10 percent of them are at risk of sight threatening retinopathy. Even if you have no symptoms, your eyes may already be showing signs of damage. Attending screening dramatically increases the chance of this damage being picked up early and treated in time. Diabetes eye screening is an *essential* part of your regular diabetes care.

To participate in screening you can ring RetinaScreen on 1800 45 45 55 or email them at info@diabeticretinascreen.ie , or complete the enclosed consent form and send it to RetinaScreen using the freepost envelope provided. If you have any further questions or if you want help, come into the practice to talk to the nurse who can help you fill out the enclosed form. I enclose a short leaflet with more information about screening. If you are currently participating in eye screening, please accept my apologies for this letter.

Sincerely,
