# Supplementary material for: Correction to: Development of an intervention to facilitate implementation and uptake of diabetic retinopathy screening
Source: Implement Sci. 2020 Jul 30;15:61. doi: 10.1186/s13012-020-01026-7 (PMC7393863; doi:10.1186/s13012-020-01026-7)
Supplement: Supplementary file 1 — Additional file 9. [file 13012_2020_1026_MOESM1_ESM.docx]

**
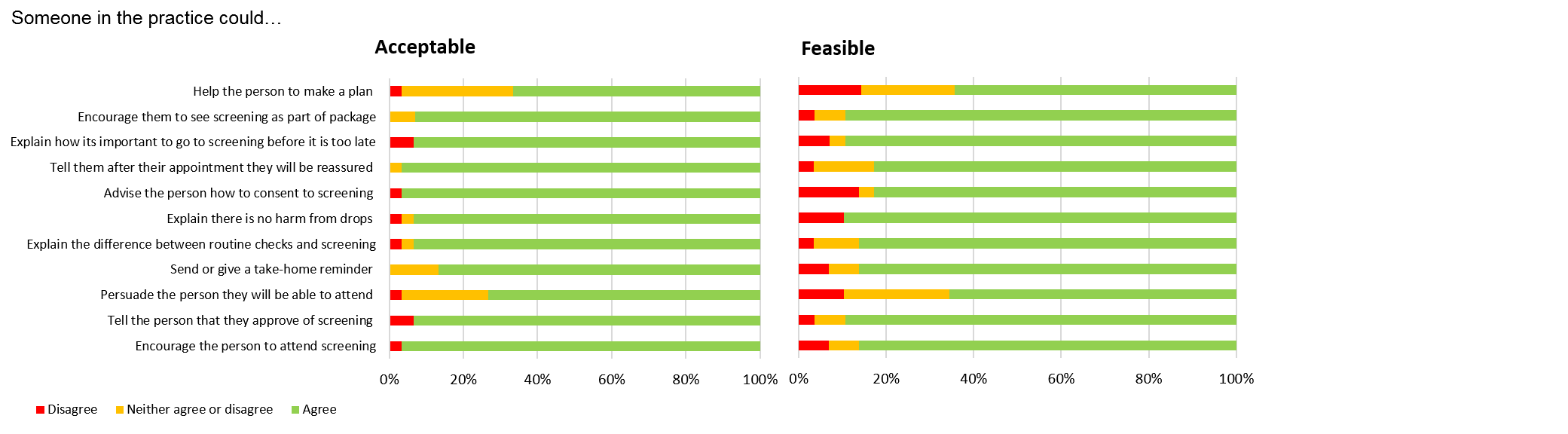
**

Suppl. Figure 1 (a) Ways to encourage patients to attend (practice led)*


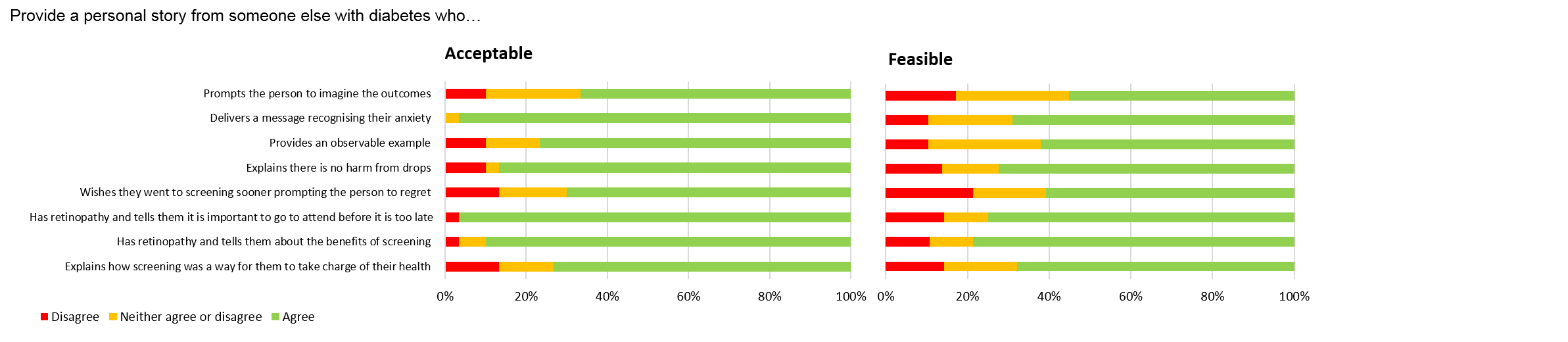


Suppl. Figure 1 (b) Ways to encourage patients to attend (narrative led)*


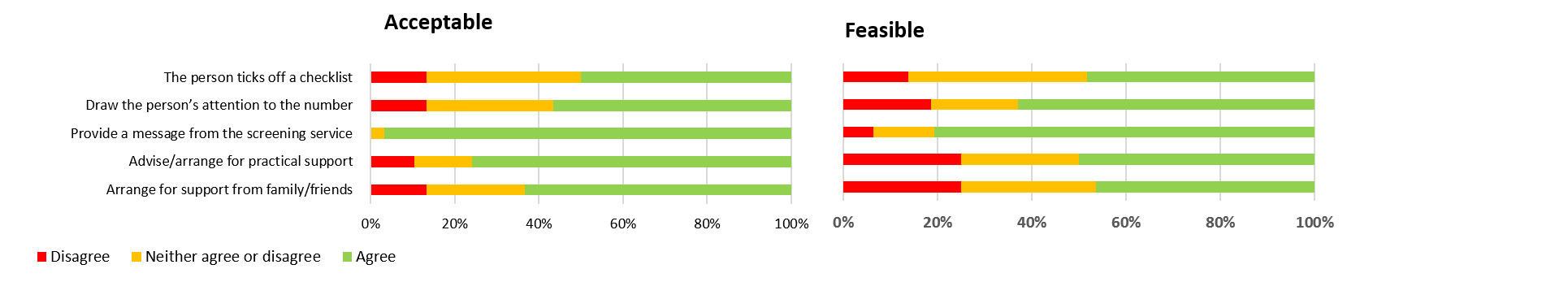


Suppl. Figure 1 (c) Ways to encourage patients to attend (other ideas)*


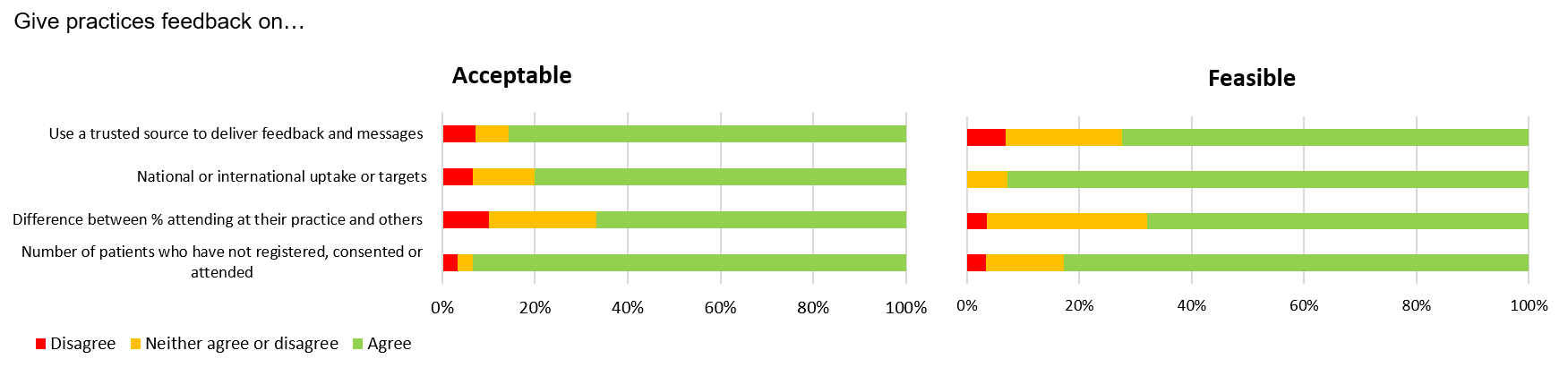


Suppl. Figure 1 (d) Ways to encourage professionals to prompt patients about screening (feedback)*


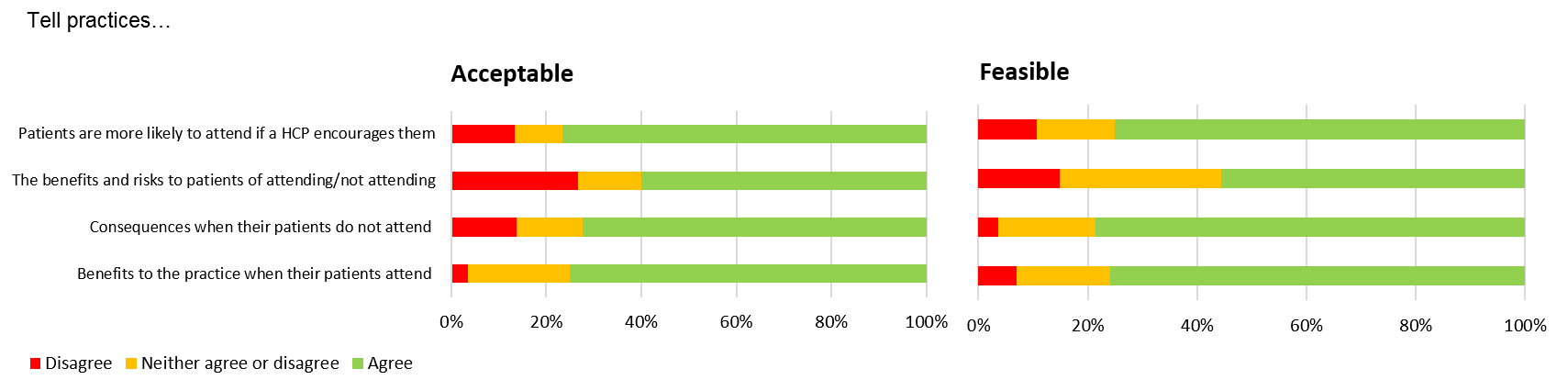


Suppl. Figure 1 (e) Ways to encourage professionals to prompt patients about screening (feedback)*


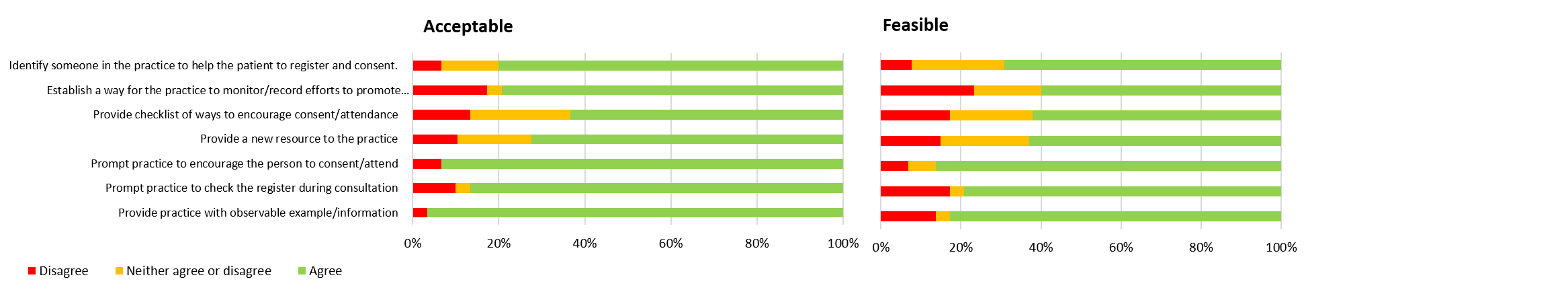


Suppl. Figure 1 (f) Ways to encourage professionals to prompt patients about screening (other ideas)*

*30 participants completed the questionnaires (13 patients and 17 health care professionals)
